# Foundational Competencies and Responsibilities of a Research Software Engineer: Current State and Suggestions for Future Directions

**DOI:** 10.12688/f1000research.157778.2

**Published:** 2025-09-08

**Authors:** Florian Goth, Renato Alves, Matthias Braun, Leyla Jael Castro, Gerasimos Chourdakis, Simon Christ, Jeremy Cohen, Stephan Druskat, Fredo Erxleben, Jean-Noël Grad, Magnus Hagdorn, Toby Hodges, Guido Juckeland, Dominic Kempf, Anna-Lena Lamprecht, Jan Linxweiler, Frank Löffler, Michele Martone, Moritz Schwarzmeier, Heidi Seibold, Jan Philipp Thiele, Harald von Waldow, Samantha Wittke

**Affiliations:** 1 Würzburg-Dresden Cluster of Excellence ct.qmat, Julius-Maximilians-Universität Würzburg, Würzburg, Bavaria, Germany; 2 European Molecular Biology Laboratory, Heidelberg, Baden-Württemberg, Germany; 3 Cluster of Excellence IntCDC, University of Stuttgart, Stuttgart, Baden-Württemberg, Germany; 4 ZB MED Information Centre for Life Sciences, Cologne, North Rhine-Westphalia, Germany; 5 Institute for Parallel and Distributed Systems, University of Stuttgart, Stuttgart, Baden-Württemberg, Germany; 6 School of Computation, Information and Technology, Technical University of Munich, Garching, Bavaria, Germany; 7 Leibniz University Hannover, Department of Cell Biology and Biophysics, Hanover, Lower Saxony, Germany; 8 Department of Computing, Imperial College London, London, England, UK; 9 Institute of Software Technology, German Aerospace Center DLR Berlin, Berlin, Berlin, Germany; 10 Helmholtz-Zentrum Dresden-Rossendorf, Dresden, Saxony, Germany; 11 Institute for Computational Physics, University of Stuttgart, Stuttgart, Baden-Württemberg, Germany; 12 Geschäftsbereich IT, Charité Universitätsmedizin Berlin, Berlin, Berlin, Germany; 13 The Carpentries, Oakland, California, USA; 14 Scientific Software Center, Universität Heidelberg, Heidelberg, Baden-Württemberg, Germany; 15 Institute of Computer Science, University of Potsdam, Potsdam, Brandenburg, Germany; 16 Technische Universität Braunschweig, Brunswick, Lower Saxony, Germany; 17 Michael Stifel Center Jena, Friedrich Schiller University Jena, Jena, Thuringia, Germany; 18 Bavarian Academy of Sciences and Humanities Leibniz Supercomputing Centre, Garching, Bavaria, Germany; 19 Mathematical Modeling and Analysis, TU Darmstadt Department of Mathematics, Darmstadt, Hesse, Germany; 20 Institute for Globally Distributed Open Research and Education, Gothenburg, Västra Götaland County, Sweden; 21 Weierstrass Institute for Applied Analysis and Stochastics, Berlin, Berlin, Germany; 22 Scientific Computing, Leibniz University Hannover Institute of Applied Mathematics, Hanover, Lower Saxony, Germany; 23 Centre for Information Management, Johann Heinrich von Thünen Institute, Braunschweig, Lower Saxony, Germany; 24 CSC IT Center for Science Ltd, Espoo, Uusimaa, Finland

**Keywords:** research software engineering, RSE, competencies, curriculum design, teaching

## Abstract

The term Research Software Engineer, or RSE, emerged a little over 10 years ago as a way to represent individuals working in the research community but focusing on software development. The term has been widely adopted and there are a number of high-level definitions of what an RSE is. However, the roles of RSEs vary depending on the institutional context they work in. At one end of the spectrum, RSE roles may look similar to a traditional research role. At the other extreme, they resemble that of a software engineer in industry. Most RSE roles inhabit the space between these two extremes. Therefore, providing a straightforward, comprehensive definition of what an RSE does and what experience, skills and competencies are required to become one is challenging. In this community paper we define the broad notion of what an RSE is, explore the different types of work they undertake, and define a list of foundational competencies as well as values that outline the general profile of an RSE. These foundational skills are encountered to a large extent within the skill sets of current RSEs in Germany and beyond, and we propose them as a starting point for aspiring RSEs to shape their technical profile. Further research and training can build upon this foundation of skills and focus on various aspects in greater detail. We expect that graduates and practitioners will have a larger and more diverse set of skills than outlined here. On this basis, we elaborate on the progression of these skills along different dimensions. We look at specific types of RSE roles, propose recommendations for organisations, give examples of future specialisations, and detail how existing curricula fit into this framework.

## Contents

Foundational Competencies and Responsibilities of a Research Software Engineer: Current State and Suggestions for Future Directions1

 [version 1; peer review: 2 approved]1

   Abstract6

 Contents6

1. Introduction9

 1.1 Terminology10

  1.1.1 The term Research Software Engineer10

  1.1.2 Further definitions11

2. Related work11

3. Values13

 3.1 Current challenges14

  3.1.1 Data security14

  3.1.2 Mentoring and diversity14

  3.1.3 Shaping digital science15

  3.1.4 Addressing environmental sustainability within planetary limits15

  3.1.5 Emerging challenges15

4. Foundational RSE competencies15

 4.1 Software/Technical skills16

  4.1.1 Classical software engineering skills16

  4.1.2 Adapting to the software life cycle ( 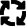 SWLC)17

  4.1.3 Creating documented code building blocks ( 
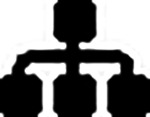
 DOCBB)17

  4.1.4 Building distributable software ( 
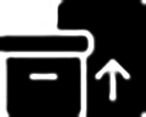
 DIST)17

  4.1.5 Use software repositories ( 
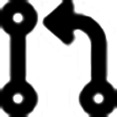
 SWREPOS)18

  4.1.6 Software behaviour awareness and analysis ( 
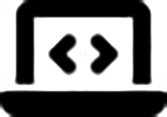
 MOD)18

 4.2 Research skills18

  4.2.1 Conducting and leading research ( 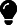 NEW)18

  4.2.2 Understanding the research cycle ( 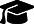 RC)18

  4.2.3 Software re-use ( 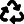 SRU)19

  4.2.4 Software publication and citation ( 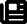 SP)19

  4.2.5 Using domain repositories/directories ( 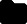 DOMREP)19

 4.3 Communication skills20

  4.3.1 Working in a team ( 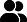 TEAM)20

  4.3.2 Teaching ( 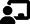 TEACH)20

  4.3.3 Project management ( 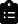 PM)20

  4.3.4 Interaction with users and other stakeholders ( 
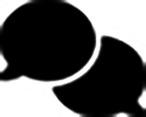
 USERS)20

 4.4 RSE tasks and responsibilities21

5. How much do different people need to know?23

 5.1 Career level23

 Table 1. Levels of technical skills expected per RSE career stage.24

 Table 2. Levels of research skills expected per RSE career stage.24

 Table 3. Levels of communication skills expected per RSE career stage.25

 5.2 Helpful RSE skills for researchers in an academic career26

 5.3 Project team structures27

 Table 4. Levels of software eng. skills expected per team structure.28

 Table 5. Levels of research skills expected per team structure.30

 Table 6. Levels of communication skills expected per team structure.32

 5.4 Designing a generalist RSE framework33

  5.4.1 An example master’s programme for research software engineering33

  5.4.2 An example of a possible career path35

6. RSE specialisations37

 6.1 Specialisations within the core RSE competencies37

 6.2 Specialisations outside the core RSE competencies38

 6.3 Existing frameworks for specialised RSE roles39

  6.3.1 Bioinformatics skills and certification39

  6.3.2 HPC skills and certification40

7. Future work41

8. Conclusion41

 Contribution details42

Ethics and consent43

Glossary43

Skill codes44

Acronyms44

Data availability statement46

Acknowledgements46

References46

  Article Information (continued)51

## 1. Introduction

Computers and software have played a key role in the research life cycle for many decades. They are now vital elements of the research process across almost all domains. They enable researchers to collect and process ever-increasing amounts of data, simulate a wide range of physical phenomena across previously unexplored scales of the universe, and discover previously inconceivably complex structures in nature and societies via machine learning (ML). This prevalence of computation and digitally-aided data analysis in research means that digital skills are now required by researchers at all career levels, and in fields significantly beyond those that would previously have been expected. Research software is now used and developed not only in science, technology, engineering and mathematics (STEM) domains, but also in other fields, like medicine and the humanities.

Researchers often lack the skills to use specialised software for their research, let alone write it. ^97^ If they come from a non-technical domain, they may also struggle to know what to ask when trying to request help from and interact with more experienced colleagues. A gap still exists in academic education, as many curricula do not sufficiently prepare students in this regard. This situation is exemplified by the extracurricular Massachusetts Institute of Technology (MIT) class “The Missing Semester of Your CS Education”, ^3^ which aims to increase “computing ecosystem literacy” even among students of Computer Science at MIT.

Researchers investing increasing amounts of their time developing their software engineering (SE) skills to support their research work can find themselves with little time to do the research itself. This, in turn, presents career development challenges since the experience required to gain and progress in research and academic roles is traditionally assessed through metrics that do not directly include software outputs. A recent shift towards the establishment of the distinct role of a *“Research Software Engineer”* ^69^ (RSE, a term that was coined in the United Kingdom (UK) a little over 10 years ago ^43^), now provides a base on which sustainable career opportunities can be (and are being) built, allowing for better training of researchers and more effective support for the development of high quality research software. There is still a long way to go, but positive change is well underway.

RSEs may work within one of the increasing number of research software engineering teams that have been set up at universities and research organisations over the past decade, or they may be embedded within a research team. They may have a job title that officially recognises them as an RSE, or they may have a standard research or technical job title such as Research Assistant, Research Fellow, or Software Engineer. Regardless of their job title, RSEs share a set of core skills that are required to design and develop research software, understand the research environment, and ensure that they produce sustainable, maintainable code that supports reproducible research outputs, following the Findability, Accessibility, Interoperability and Reusability (FAIR) principles. ^5^

This community paper defines a set of core values and foundational competencies, which an RSE should acquire during training and formal education. These skills are formulated independently of a specific research domain and current technical tools used to support the application of the skills. By defining these competencies, we provide a guiding framework to facilitate the training and continuous professional development of RSEs, thus helping to provide a positive impact on research outputs and, ultimately, society as a whole. These competencies draw upon skills from traditional SE practice, established research culture, and the commitment to being part of a team. However, we see this set of skills as a foundation to build upon. We envision that through specialised training, the set of skills of graduate RSEs and domain researchers will grow. This is underlined by a growing interest to perform RSE research, i.e. research into methods and tools more catered to the unique challenges that research software provides.

While this community paper is based on workshop discussions that were attended largely by RSEs (deRSE23 in Paderborn, ^37^ un-deRSE23 in Jena, and deRSE24 in Würzburg, all in Germany), we believe that the competencies formulated here can offer far-reaching impact beyond the domain of RSE into adjacent aspects of research and, indeed, the wider research community. This is especially important given that much research involves some amount of data management, processing and visualisation, or the creation of tools for these tasks, and funding bodies and computing infrastructure providers will sometimes prioritise projects that generate archived, annotated, re-usable, and potentially remotely executable data. In particular, funding agencies and research managers will find the discussion in this paper valuable in order to discover where RSEs see their place in the existing landscape of scientific domains and how to support the work of RSEs at different positions and career levels. While we draw mostly from experiences of RSEs working in Germany or, in some cases, across Europe and the US, our recommendations do not focus on a particular region.

The outline of the paper is as follows. We start with a non-exhaustive overview of existing initiatives in Section 2. Section 3 elaborates on the values that provide the guiding principles for the work of an RSE. Section 4 defines a set of core skills based on these values. We categorise these skills into three pillars, namely “software/technical”, “research”, and “communication” skills, reflecting the hybrid nature of an RSE. To justify the selection of these skills, we also list some current tasks and discuss the skills used therein. As with any general skill set, not all RSEs will need to use all the skills highlighted to the same level of expertise. Therefore, Section 5 examines how much a person needs to know depending on their education or career level or on the type of projects they would like to be involved with. In the same section, we provide an overview of what skills and limitations an RSE in different team structures typically has, and we give recommendations for organisations that need to support RSEs. Section 6 provides a list of RSE specialisations and discusses the level of skill needed to work in each of them, before we conclude the paper with details of future work in Section 7 and conclusions in Section 8.

### 1.1 Terminology

**1.1.1 The term Research Software Engineer**

Research Software Engineering can be considered an interface discipline, linking traditional Software Engineering with Research itself. ^57^ Due to this nature there is a plethora of different variations of RSE depending on the particular Research domain they are working in. Therefore the broad notion of Research Software Engineers is better thought of as a collection of sub-communities. The term Research Software Engineer is made more difficult to grasp since an internationally recognised definition is still missing. While there is consensus about the general notion that an RSE is a person with one leg in their research domain and the other in software development, this spans a whole spectrum depending on which one is more emphasised. There is also the question of what level of professionalism concerning both non-SE research and SE is expected. A more inclusive definition allows more people to self-identify as RSEs, thereby also fostering an inclusive community of people working in digital science (see also Section 3 on the values of an RSE). RSEs fall therefore somewhere on the spectrum between a researcher at one end and a software engineer at the other. Common to all of them is, that they need to be able to work in the research environment the software is used in, ideally at eye-level with native researchers, but at least as close as possible. RSEs often need to deal with non-technical complexities that are characteristic for research environments: organisational, motivational, with respect to the size of projects, independence and heterogeneous goals of stakeholders, boundary conditions for funding and future funding, to name just a few. Summarising, RSEs have skills and experience in three important areas: in the research area(s) their software is used in, in software engineering topics, as well as in interdisciplinary communication.

**1.1.2 Further definitions**

Depending on the national research environments and processes that readers are familiar with, the notion of the terms *software* and *research* might differ. Therefore, to avoid ambiguities, we define these as follows:

**Software**: Source code, documentation, tests, executables and all other artefacts that are created during the development process that are necessary to understand its purpose.

**Research software**: Foundational algorithms, the software itself, as well as scripts and computational workflows that were created during the research process or for a research purpose, across all domains of research. This definition is broader than in ^5^ and is the outcome of a recent discussion in. ^40^

**Research software engineers**: People who create or improve research software and/or the structures that the software interacts with in the computational environment of a research domain. They are highly skilled team members who may also choose to conduct their own research as part of their role. However, we also recognise that many RSEs have chosen specifically to focus on a technical role as an alternative to a traditional research role because they enjoy and wish to focus on the development of research software.

**Researchers**: People who are using the services provided by Research Software Engineers. This, on purpose, is a very broad definition and was chosen for a better reading.

## 2. Related work

Various initiatives are working to support technical professionals develop their computational skills. Particularly related to this work are initiatives that aim to define sets of such skills and to guide the community with certification programs and training resources.

**RSE Competencies Toolkit:** The RSE Competencies Toolkit ^75^ is a community project that developed out of a hack day activity at the 2023 edition of the annual Software Sustainability Institute Collaborations Workshop. ^102^ The toolkit provides a web application that aims to support technical professionals in understanding how to develop their skills. It enables them to build a profile of their competencies within the system, while it also provides a set of training resources that are linked to a competency framework.

**HPC Certification Forum:** The High-Performance Computing (HPC) Certification Forum ^88^ is working towards providing a certification process for HPC skills. As part of this process, the group is developing a Competence Standard ^89^ and an associated skill tree that provides a classification of HPC competencies. This work aims to develop a standardised representation of relevant HPC knowledge and skills which can, in turn, lead to structured and recognised sets of skills that can underpin the certification process.

**EMBL-EBI Competency Hub:** The European Molecular Biology Laboratory - European Bioinformatics Institute (EMBL-EBI) Competency Hub ^25^ provides a bioinformatics/computational biology-focused example of a competency portal. In addition to collecting information on a range of competencies that can be browsed within the web-based tool, it also provides career profiles for roles within the domains that EMBL-EBI focuses on. The hub provides access to a variety of training resources that are linked to the specific competencies that they relate to. This enables learners to more easily find the right training materials in order to support their career development journey, helping them to identify what they might want to learn and in what order.

**Training-focused initiatives:** Further initiatives implicitly define sets of competencies by providing (open) teaching material for selected skills. This is a non-exhaustive list of related initiatives, which will be discussed in more detail in a separate publication. In some cases, the activities extend beyond training, but they do not focus on defining frameworks of competencies.

One prominent example is the Carpentries, ^85^ a non-profit entity that supports a range of open source training materials and international communities of volunteer instructors and helpers who run courses around these materials. A similar framework is provided by CodeRefinery, ^18^ currently funded by the Nordic e-Infrastructure, as well as SURESOFT, ^8^^,^^103^ a project at Technical University (TU) Braunschweig and Friedrich-Alexander-University (FAU) Erlangen-Nürnberg, funded by the German Research Foundation (DFG, Deutsche Forschungsgemeinschaft) and targeting more advanced SE topics such as software design principles, design patterns, refactoring, continuous integration (CI) and test-driven development (TDD). The INTERSECT RSE Training project ^14^^,^^50^ also provides training materials and organises training events in the USA, funded by the NSF.

There are also several initiatives focused on training HPC-oriented RSEs, such as the Partnership for Advanced Computing in Europe (PRACE) ^72^ (with material aggregated on various websites, e.g., on EuroCC Training ^26^), Understanding and Nurturing an Integrated Vision for Education in RSE and HPC (UNIVERSE-HPC) ^93^ (a project funded under the UK’s ExCALIBUR research programme ^30^), and the EuroCC National Competence Center Sweden (ENCCS), ^28^ which offers a collection of lessons for HPC skills. ^27^ At the intersection between HPC and the broader RSE field, the IDEAS PRODUCTIVITY project ^47^ organises online events, provides training material via the Better Scientific Software (BSSw) project ^6^ and maintains HPC-focused guidelines, such as the Extreme-scale Scientific Software Development Kit. ^100^

Initiatives focused on Germany include EduTrain ^104^ (a section of the National Research Data Infrastructure (Nationale Forschungsdateninfrastruktur) (NFDI) ^105^), the Helmholtz Federated IT Services (HIFIS), ^41^ and the already mentioned SURESOFT. ^103^

## 3. Values

It is important that the activities of an RSE are guided by ethical values. In addition to the values for good scientific practice, ^33^ RSEs also need to adhere to the SE Code of Ethics. ^38^ Central to that code is the RSE’s obligation to In addition to the values for good scientific practice commit to the health, safety and welfare of the public and act in the interest of society, their employer and their clients. Further values loosely based on that code include the obligations

• to commit to objectivity and fact-based, honest research conclusions,
• to promote openness and accountability in the research process,
• to take great care to develop software that adheres to current best practices,
• to judge independently and maintain professional integrity,
• to treat colleagues and collaborators with respect and work towards a fair and inclusive environment, and
• to promote these values whenever possible and make sure that they are passed on to new practitioners.

Many practitioners will follow the values expressed in these codes of conduct without knowing them because they are passed on implicitly by their peers and mentors. ^19^ Here, they are stated explicitly because they underpin the foundational competencies and responsibilities of RSEs who are professionals living in both worlds.

The deployment of computer-based modelling and simulation has dramatically changed the practice of science in a large number of fields. It has enabled the hitherto impossible study of new classes of problems, often replacing traditional experimentation and observation (it can also serve to integrate a communal body of knowledge). ^71^ Thereby it has the potential of changing our way of generating knowledge, while at the same time it challenges our notions of explaining science. Humphreys ^46^ regards this development as “more important than the invention of calculus in the 1660s, an event that remained unparalleled for almost 300 years”. The epistemological status of computer modelling and simulation is still the subject of debate, which ranges from the postulate of a new process of knowledge creation that has its own, unique, epistemology ^99^ to the perception that from a philosophy of science perspective, there is nothing really new. ^31^ In any case, it is clear that a number of decisions in the construction of a simulation-model will have a significant impact on the adequacy for purpose ^9^ of the model. These decisions include the selection of the salient characteristics of the system to be modelled, the choice of the mathematical representation of the processes to be represented, the choice of numerical methods and other algorithms and even including the design of the user-interface.

The relationship between initial state, inputs and final state of a computer simulation is “epistemically opaque”, ^46^ in that not every step of the process is directly observable. The current trend of an increasing application of computationally irreducible systems, such as those based on artificial neural networks, further exacerbates this inherent limitation of explainability. An RSE usually takes a pivotal role in assessing this adequacy for purpose of a model as well as in characterising and communicating the domain of its legitimate application and its limits of interpretability. This role, together with the enormous reliance on modelling and simulation of scientific results, as well as real-world decision-making, places a large responsibility on the RSE. It is important that RSEs are aware of this responsibility and continuously improve their capabilities to live up to it.

Research software is also well on its way to being ever-present in data-driven research, in all research fields. This can probably be most prominently seen by considering software used to analyse data, e.g. within experimental research. It is not unusual for RSEs to support those more research data oriented efforts as well. Here, specifically, they closely interact with research data management professionals and practices by designing research software that is better able to adhere to the FAIR principles for research data, but also to follow similar rules for research software (FAIR4RS ^5^). As such, they are then familiar with special requirements stemming from the field itself, e.g., in medical research, and with privacy related issues especially for personal data, e.g., for conducting surveys.

RSEs often assume a multifaceted role at the junction of research, SE and data management. They work with a varying and diverse set of colleagues that might include other developers, support unit staff and academics of different fields and all career stages. This situation yields a specific set of challenges RSEs should be aware of to consciously make ethically sound judgement calls. Below we list some example areas that highlight present-day challenges.

### 3.1 Current challenges

**3.1.1 Data security**

A lot of RSE work involves the manipulation or creation of data processing tools. We highlight that professional conduct requires these creations to be reliable and to maintain data integrity. In particular, the way that personal data is handled can have far-reaching implications for society. Independent of the encoding into the respective national law in an RSE’s jurisdiction, the right to information privacy is internationally recognised as a fundamental human right, e.g., in the European Convention on Human Rights. ^20, 45^ RSEs need to be aware of this topic’s importance and deal with tensions that might arise with researchers’ desire for trouble-free sharing of data, thereby expecting openness about the research process, versus the integrity expectations of the society towards information technology (IT) systems. Handling personal data also has ramifications for information security considerations during the software development process. Data protection is a complex topic, so RSEs should be aware that they may need to consult external expertise, for example when dealing with special topics such as cryptography or re-identification attacks. ^42^

**3.1.2 Mentoring and diversity**

RSEs are often experienced professionals who work closely with and provide technical training and guidance to early career researchers. Similarly to academic supervisors, they bear a certain responsibility to guide and advise less-experienced colleagues with respect to career development and the achievement of academic goals. This can take the form of supervising a student or mentoring a fellow RSE. The RSE needs to be aware of the biases arising from the sociological imbalances in research and academia. According to the United Nations Educational, Scientific and Cultural Organization (UNESCO) Science Report ^78^ women account for 33.3% of all researchers. 60.2% of researchers come from high-income countries which account for 17.5% of the global population in 2018. Furthermore, the socioeconomic background of academics is not representative of the general population, for example in the US a tenure-track academic is 25 times more likely to have a parent with a PhD. ^65^ Thereby, to promote their values of an honest, open, and inclusive research space, they should be aware of the diversity problems and help to mitigate them whenever they have the chance to do so.

**3.1.3 Shaping digital science**

Through writing research software, RSEs hold an important role in the process of scientific production. Their choices might determine whether the respective research is reproducible or not, whether the results can be re-used, whether future research can build on existing tools or has to start from scratch. Builders of larger research-infrastructure projects determine to some extent the possibilities and limitations of future research and therefore need to be able to make a value-based judgement on topics such as open science, path dependence, and vendor lock-in.

**3.1.4 Addressing environmental sustainability within planetary limits**

The last two decades saw transistor technology approach the limits of attainable miniaturisation, and maximum chip clock frequency begin to plateau. ^84^ Nevertheless, a misleading belief in limitless growth of computing capabilities (storage, computing power, transfer speed) is still widespread within popular perception. A practical consequence of this is an ever-growing demand for resources to cover the expanding need of storage and processing, with no clear deceleration in sight (e.g. the IEA estimates a doubling in data centres energy consumption from 2024 to 2026 ^48^). At the same time, current science is well aware of several planetary boundaries being exceeded due to human activities. ^74^ Data processing, storage and transfer account for a non-negligible fraction. ^48^ Demands to move resource consumption to a sustainable rate are well justified and supported by science. ^81^

RSEs have the opportunity to contribute to this effort by, for example, choosing computationally adequate approaches (e.g. recognising where a proven statistical method may suffice in place of a power-hungry AI model, or configuring a test pipeline to minimise redundancy), and embracing data frugality measures (e.g. recognising sufficient resolution when sampling data for processing or storage). If past computational solutions were frugal because of technological limits, in future they should tend to that by virtue of an awareness of what may be adequate. The Governance, Responsibility, Estimation, Energy and embodied impacts, New collaborations, Education and Research (GREENER) principles ^59^ suggest how these concerns can be addressed and how research computing can become more environmentally sustainable.

**3.1.5 Emerging challenges**

RSEs often operate at the cutting edge of technological development and therefore might have to deal with technologies of which the dangers and drawbacks are still poorly understood. A current example is the rush for the application of large language models (LLMs), where RSEs working in these fields should stay up-to-date and be able to help researchers assess topics such as training-data bias, LLM “hallucinations” or malicious use, with the greater goal of making these powerful tools work for the welfare of society.

## 4. Foundational RSE competencies

The role of an RSE lies somewhere on the spectrum between that of a researcher (the “R”) and a software engineer (the “SE”) and, therefore, requires competencies in both fields. RSEs typically have a background in research or software engineering, but they definitely have obtained broader knowledge in both fields. Even when working as the only RSE on a task or project, they typically apply their knowledge and experience as part of larger teams of researchers and technical professionals, which allows them to cultivate this hybrid nature. There are many ways to categorise the competencies of an RSE. We chose to distribute these competencies over three pillars to reflect the fact that RSEs are both competent researchers (the research skills, Section 4.2) and software engineers (the software/technical skills, Section 4.1). The third pillar (communication skills, Section 4.3) forms the bridge between the former two categories, with a particular focus on the software and research cycle and the scientific process. These competencies are relevant in a broad setting and form the foundation for specific specialisations. These competencies have been chosen in order to make RSEs contribute to an open and inclusive research environment, with tools that respect their professional values (see Section 3).

These skills and competencies come into play in various forms: The RSEs themselves need to acquire and develop them as their career progresses ( **Career level**). However, some knowledge of software and data processing is required at all academic levels and for all positions ( **Academic Progression**). The relative importance of the skills and competencies also depends on the size of the RSE team ( **Project team size**). Finally, different sets of skills are emphasised in the different RSE specialisations ( **RSE specialisations**).

During the Paderborn workshop (deRSE23) we asked learners and novice RSEs what they would like to have learnt. The top five items mentioned were (Goth et al. ^37^): testing, contributing to large projects, when or why to keep repositories private, high-quality software development, and finding a community. Those topics comprise combinations of the skills and competencies defined below. We will elaborate these in Section 4.4.

### 4.1 Software/Technical skills

Besides skilled researchers, RSEs are also competent software engineers. As such, they ideally can solve complex software engineering problems and design software as a user-oriented, future-proof product. The technical skills required by an RSE overlap to a large extent with the common fundamental software engineering skills (see, e.g., Landwehr et al. ^58^), but put greater emphasis on aspects related to achieving good scientific practice and to serving special needs of research software. In addition, a lot of RSEs are either self- or peer taught in these skills (see, e.g., figure 14 in Barker et al. ^4^). These skills include requirements analysis, design, construction, testing, program analysis, and maintenance of software. On the other hand, RSEs also know how to make research software adhere to the FAIR principles, ^5^ and how to achieve different levels of research software reusability (see, e.g., Chue Hong ^17^), while they have deeper understanding of the scientific context around the research software projects they work on. To reflect this, the technical skills listed below complement competencies regarding the standard life cycle of software development (as summarised in Section 4.1.1) with RSE-specific focus skills.

**4.1.1 Classical software engineering skills**

To summarise the vast range of the skills a software engineer is typically equipped with, we refer to the Guide to the Software Engineering Body of Knowledge (Bourque, Fairley, and IEEE Computer Society ^10^). Because research software engineering is an interface discipline, RSEs are often stronger in topics more commonly encountered in research software contexts (e.g., mathematical and engineering foundations) than in other areas (e.g., software engineering economics). However, they bring a solid level of competence in all software engineering topics. Therefore, RSEs can set and analyse software requirements in the context of open-ended, question-driven research. They can design software so that it can sustainably grow, often in an environment of rapid turnover of contributors. They are competent in implementing solutions themselves in a wide range of technologies fit for different scientific applications. They can formulate and implement various types of tests, they can independently maintain software and automate operations of the integration and release process. They can provide working, scalable, and future-proof solutions in a professional context and with common project and software management techniques, adapted to the needs of the research environment. Finally, as people who have often gained significant research experience in a particular discipline, they combine the necessary foundations from their domain with software engineering skills to develop complex software.

**4.1.2 Adapting to the software life cycle (** 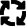 **SWLC)**

The traditional software development life cycle defines the stages that form the process of building a piece of software. Initial development generally involves an analytic process where requirements and ideas are gathered and analysed (requirements engineering), followed by formulating a plan to fulfil them (design) that is finally turned into running code (implementation). This is accompanied by different measures of quality control (e.g., reviews, testing), validating and verifying that things work as expected and that they continue to do so when development progresses further. Depending on the software project, this can mean a simple “Think-before-you-do”, or more elaborate and formal processes. ^67^ Often the development cycles are executed iteratively and incrementally. The life cycle further includes periods of deployment, maintenance and further development (software evolution), as well as software retirement. To assess the current state and needs of the software, the RSE should be familiar with different maturity metrics, e.g. the DLR application classes, ^77^ the research software maturity model ^21^ or technology readiness levels (TRLs). Additionally, the research software life cycle extends the traditional life cycle with software publication. The RSE should be aware of this life cycle and be able to predict and cater to the changing needs of a software project as it moves through the stages.

**4.1.3 Creating documented code building blocks (** 
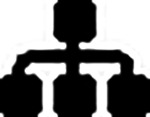
 **DOCBB)**

The RSE should be able to create building blocks from source code that are reusable. This ranges from simple libraries of functions up to complex architectures consisting of multiple software packages. An important part of enabling code reusability is the provision of sufficient information in the form of comments within code, documentation or other means. This is vital to ensure that developers and maintainers understand what a piece of software aims to do and how to enable others to use the provided functionality. This is primarily achieved through a “clean” implementation and enhanced by documentation. Documentation ranges from commenting code blocks to using documentation (building) tools. It should be written with consideration for the different audiences who may need it depending on their goals and expertise, for example by following the Diátaxis framework. ^73^

**4.1.4 Building distributable software (** 
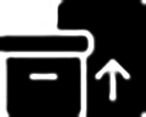
 **DIST)**

The RSE should be able to distribute their code on their domain/language specific distribution platforms. This almost always encompasses handling/documenting dependencies with other packages/libraries. It sometimes requires knowledge of using build or package management systems to enable interoperability with other projects. In terms of usability and needs of the user community the RSE should be able to decide whether a library or a framework is the right type of program to build and distribute.

**4.1.5 Use software repositories (** 
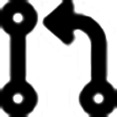
 **SWREPOS)**

The RSE should be able to identify and use fitting software Forges (often just termed “repos”) to share the artefacts they have created and, if possible, invite the public to scrutinise them in an open review process. These software repositories usually provide facilities for software development, which differentiate them from the domain repositories described later.

**4.1.6 Software behaviour awareness and analysis (** 
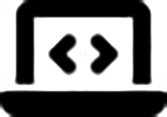
 **MOD)**

We define this as a certain quality of analytical thinking that enables an RSE to form a mental model of a piece of software in a specific environment (program comprehension). Using that, an RSE should be able to make predictions about a software’s behaviour. This is a required skill for common tasks such as debugging, profiling, optimising, designing good tests, or predicting user interaction. Many tools exist to help with understanding and evaluating existing code, especially from a structural point of view. An RSE should understand their output and its implications. An important facet of this capability relates to information security. RSEs need to consider the safety and integrity of personal data and other sensitive information and make sure that they do not negatively impact the integrity of their institution’s network and computing infrastructure (see Section 3.1.1).

### 4.2 Research skills

**4.2.1 Conducting and leading research (** 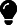 **NEW)**

RSEs are curious and able to conduct research, both on research software engineering, and on their research-wise “home domain” (see also Section 5.4.1). Senior RSEs are also able to lead research, and many RSEs have a doctorate. ^44^ Since RSEs often operate in different research fields, they also gain their reputation from their effectiveness in interacting with researchers from the same or other domains. Therefore, some curiosity together with a broad overview of the research field is required, as this enables the RSE to learn new methods and algorithms directly from domain peers. Similarly, a broad overview of the field of SE research and the growing field of RSE research enables the RSE to learn, apply, and teach new methods and tools for improving the way they develop software. This curiosity, together with the ability to convert it into new ideas, is also reflected when an RSE is actively trying out new tools or discovering related literature from adjacent domains. Lifelong learning is then no longer just a phrase but becomes a motivation to work.

**4.2.2 Understanding the research cycle (** 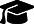 **RC)**

One of the key skills that RSEs have is their understanding of how research works. They embrace being part of a larger community which, despite friendly competition, shares the common goal of gaining knowledge to disseminate it. Thereby they know that they are part of a bigger undertaking that involves many other parties in and outside their domain, and also that their software can be utilised at different stages of the research cycle by different people. They may be asked to contribute to the ethical and regulatory evaluation of a project to ensure integrity of the research performed therein. Like other researchers, RSEs are open to discussions and arguments beyond their own expertise and appreciate the underlying principles of good research, including publications, reviews and reproducibility.

**4.2.3 Software re-use (** 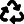 **SRU)**

The re-use of existing assets such as libraries and pieces of code to improve efficiency and quality belongs to the fundamentals of software construction. ^10^ To discover software, RSEs rely on domain-specific knowledge and domain repositories, as well as research skills, discovering related software via software citations and metadata. To evaluate whether the artefacts to be re-used suit their needs, RSEs often need to consider the scientific context of their origin. For example, a paper that references the code under consideration might be crucial to validate its fitness for purpose or lack of suitability. Code that incorporates research-domain specific knowledge needs to be understood at a very detailed level and its re-use documented to meet standards of good research practice. Not only the technical compatibility needs to be understood and documented (programming languages, system interoperability), but also the underlying models and computational methods need to fit the purpose; this question often requires wider research skills and deeper understanding of the research domain at hand.

**4.2.4 Software publication and citation (** 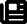 **SP)**

Another part of FAIR software is concerned with publishing new and derived works and making them available for re-use by the research community and the general public, within the boundaries set by their institutional policies. RSEs need to have a basic understanding of common software licence types, including proprietary and open source licences and how “copyleft” and “permissive” open source licences differ. They should also understand compatibility between different licences, and the ramifications for re-using and composing programs. Beyond that, RSEs will need to properly execute the technicalities of software publishing. These include the application of licences and copyright statements, understanding and assigning software authorship, crediting contributors, maintaining FAIR software metadata and publishing software artefacts on respective publication platforms. Finally, RSEs will need to understand the principles of software citation. ^82^ This concerns both the potential for reuse of their own work, which demands the provision of complete and correct up-to-date citation metadata for their software, as well as their own citation obligations deriving from building on previous work in the form of dependencies.

**4.2.5 Using domain repositories/directories (** 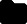 **DOMREP)**

Almost all research software is developed within a specific scientific domain. Some software may be able to cross boundaries, but the majority will have a home domain, with which it needs to be able to interact. The RSE then needs to be aware of any domain specific repositories that will contain data sets, catalogues, and other domain specific artefacts, in addition to software. The RSE also needs to be aware of how their software can interact with the existing domain-specific data repositories. Finally, they need to be able to assess and use software repositories - domain-specific or generic - for publishing software with the relevant metadata.

### 4.3 Communication skills

RSEs do not work in isolation. They are embedded in a research group or work within a team of RSEs supporting particular research projects. RSEs often need to interact with and facilitate communication among colleagues, clients and contractors with a very broad spectrum of background-knowledge, specialisation, expectations, and experience whilst keeping diversity issues in mind ( Section 3.1.2). Communication skills are therefore crucially important. Team skills are also mentioned in common guides for SE such as the software engineering body of knowledge. ^10^ However, the interpersonal and organisational skills and the capacity for adaption required to work in a research setting warrants a much stronger emphasis on this field of competence.

**4.3.1 Working in a team (** 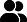 **TEAM)**

Being able to work, and effectively communicate in teams is essential for RSEs. For example, RSEs need to be able to explain particular implementation choices made and may even need to defend them. Within a team of RSEs, code reviews improve knowledge transfer and increase team cohesion. The team might change on a project-to-project basis and might be comprised of colleagues with very different backgrounds including, for example, IT staff, domain scientists and technicians working alongside software engineers. The shared values come into play and each RSE needs to ensure that these values are lived by and passed on to others. Senior RSEs may lead a team of RSEs.

**4.3.2 Teaching (** 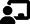 **TEACH)**

RSEs have many opportunities to teach. These range from inducting new colleagues to teaching digital skills either through short courses, for example from The Carpentries, ^85^ or entire lecture series. RSEs may also act as mentors and consultants. Code review also includes aspects of the teaching skill.

**4.3.3 Project management (** 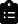 **PM)**

The RSE should have knowledge of project management processes. At some institutes, project management tools and approaches differ between individual research groups, but it is useful if an RSE understands general structures of a 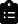 PM scheme, or can bring in new ideas for improvement. Project management in research software engineering poses specific challenges (see 
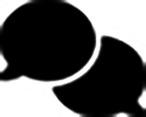
 USERS) that might require the capacity to adapt to changing conditions and deviate from common project management methods. Additionally, the RSE should know that SE offers various methods and approaches specifically tailored to management of software projects and products.

**4.3.4 Interaction with users and other stakeholders (** 
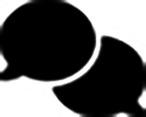
 **USERS)**

Since research software is often developed as part of the research process itself, its requirements and specifications might change with the progression of research. Stakeholders of research software often change across different research projects or even within the course of one project. Roles in connection with research software are often in flux and diffuse. For example, a single person might be user, developer and project manager at the same time. Often this means it is necessary for an RSE to think “outside their comfort zone”, but at the same time to be able to convey their knowledge and experience to experts of other fields or persons at different hierarchy levels in a way they can understand more easily. These conditions pose specific challenges for requirements analysis, project management, training and support.

### 4.4 RSE tasks and responsibilities

These skills, while already numerous are also generic on purpose. They span a multidimensional space in which the day-to-day tasks and responsibilities of an RSE can be found. We describe here some examples of the competencies applied in combination to the set of current common tasks and challenges for RSEs identified during the deRSE23 Paderborn workshop.

The most obvious task of an RSE is to develop software that is used in research. This broad topic requires all the SE skills. Of course, these are the competencies that are the most fluid since they have to adapt to frequent technological advancements. Additionally, proper SE skills often require knowledge of 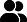 TEAM, and 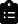 PM. Today, this means effective use of integrated development environments (IDE), static analysis tools, design patterns and documentation (for oneself and others).

The RSE needs to be able to formulate and discuss structural and behavioural aspects of software on a more general level than through the code itself and often even before a first line of code is written. A set of tools and diagrams for effective and standardised communication about software on a meta level is provided by the Unified Modelling Languages (UMLs). As a modelling tool, it is directly related to 
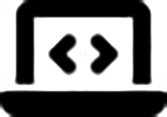
 MOD. Additionally, it can be applied in various stages of the 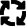 SWLC, especially in the early stages, as a first documentation of the planned modular structure to facilitate 
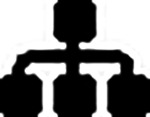
 DOCBB, 
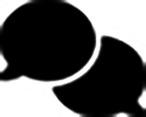
 USERS, 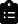 PM and 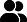 TEAM.

The RSE needs to be able to choose appropriate algorithms and techniques ( 
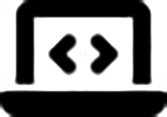
 MOD and 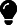 NEW). Apart from the technical feasibility, this choice is also informed by the values outlined in Section 3. For example, the RSE needs to be able to estimate resource usage (processing, memory and storage consumption, e.g. Ref. 60). Resource usage has not only a direct financial price tag but also environmental costs via associated energy consumption (see Section 3.1.4).

Software development also includes testing. This task is a manifestation of the SE competencies of 
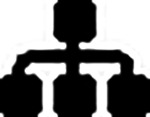
 DOCBB and 
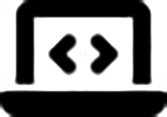
 MOD since a model of the software is required in order to write good tests that facilitate understanding and documentation. Today this encompasses the knowledge of testing frameworks as well as continuous integration and continuous delivery (CI/CD) practices. In addition to being tested, software should also provide reproducible outputs. Projects like ReproHack ^101^ can greatly help in fostering that competency.

Apart from testing, there are many code analysis tools to monitor and improve the quality of code. An RSE should be familiar with the tools available for their specific environment and how to include some of them into a CI/CD pipeline. Typically, this includes linters and similar static tools as well as dynamic tools like profilers and code coverage analysis. The development of these tools is very dynamic and environment specific. A good introduction can be found in Ref. 10 and an online resource is Ref. 106. As these tools help with behavioural and structural analysis and therefore modularisation these tools enable 
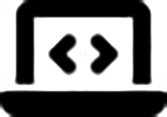
 MOD as well as 
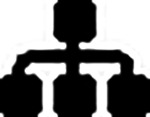
 DOCBB.

Part of the FAIR principles is to make software findable and reusable. The RSE needs to be able to decide when and why to keep a repository private. This decision requires knowledge in 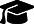 RC, 
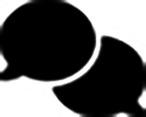
 USERS, 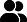 TEAM, and sometimes 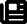 SP. Furthermore, knowledge of the practices and contractual regulations of the RSE’s institution is also required. The RSE also needs to understand metadata for research data and research software. There are ongoing efforts on metadata for research software such as CodeMeta ^51^ and the National Research Data Infrastructure (NFDI, Nationale Forschungsdateninfrastruktur) working group ^16^ on the subject. These are complemented by the development of new tools and methods for providing and working with software metadata, such as the Citation File Format project ^24^ and HERMES. ^23^ Other efforts focus on Software Management Plans (e.g., Refs. 1, 63) which could be helpful for RSEs at early stages (i.e., with not much experience of project management). They give quick hints on what to look for regarding basic management for research software (including information on, e.g., licenses, releases, publication, citation, archiving) together with some ongoing work on corresponding metadata. ^35^ Metadata can also be used actively during and within a research project, to inform the decision-making processes. ^7^

Most RSEs will contribute to other projects, some of which will be large. This is a topic that requires competency in 
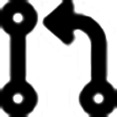
 SWREPOS, 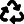 SRU, and 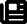 SP in order to understand the ramifications of sharing, and in 
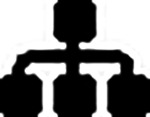
 DOCBB, since the contributed code has to be understood by others. Interacting with project members depends on the 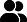 TEAM skill. Today, this frequently involves the effective use of collaborative platforms like GitHub/GitLab, honouring a project’s code of conduct, and some knowledge of popular open source software licences, e.g. the GNU General Public License (GPL).

The 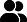 TEAM skill will play a major role when an RSE is introduced to an existing project. An existing project will have grown some idiosyncratic habits and processes. Often it will require all the skills and patience of an RSE to steer a project towards best software engineering practices, while not having a leadership position.

RSEs are embedded in communities. There are two different aspects to finding these communities: First, we have the aspect of community building for a research project. Since this deals with software that is supposed to be used in research this requires knowledge of 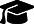 RC, 
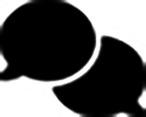
 USERS, and also 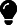 NEW, in order to effectively interact with domain scientists. Today, an example is a presence on social media. The other 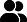 TEAM-related aspect is the embedding of recently-trained RSEs into the Research Software Engineering community, sharing the same set of values and competencies. We envision newcomers to the RSE field becoming part of a strong network of RSEs, tool-related communities, and the classical domain communities, making them more effective at supporting research. These networks are a lifelong manifestation where RSEs work to provide an inclusive environment for their peers and provide opportunities for lifelong learning.

An ever-growing list of national associations can be found at Ref. 76.

RSEs are also mentoring colleagues (see also Section 3.1.2). This necessitates giving good advice that fits to a project’s stage in its life cycle, thereby requiring knowledge of ( 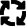 SWLC), and its context in its research domain and thus ( 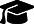 RC). Research software can often start out as a tool to answer a personal research question, becoming more important when other researchers start to rely on it. At the other end of the scale, research software can sometimes underpin key processes that deal with critical questions such as weather forecasting or medical diagnosis. A classification of software is commonly used to formalise the process of giving good advice ^77, 94^ where research software can move from one class to another during its life cycle. ^77^ Classifies applications based on their scope and criticality and provides SE recommendations. The RSE needs to be able to identify the application class they are dealing with and apply the respective RSE practices.

Often RSEs, especially in RSE groups, will develop applications and services with different variants for different research purposes and groups. Additionally, many research groups develop their own codes for specific research purposes, e.g. simulation codes or specialised data analysis pipelines. A lot of their development of new features is project-based, often through PhD projects. Work can sometimes result in code that diverges from the main project into a separate variant with re-integration planned as a final step. To reduce the chance of variant source code diverging significantly and producing a large integration overhead, 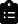 PM skills and methods are needed. More specifically, software product line management methods have been developed for this exact problem and purpose.

## 5. How much do different people need to know?

Now that we have the different competencies, we can explore various dimensions of these competencies, depending on their circumstances. A strong beneficiary of specialised RSEs can also be newly formed RSE centres at research institutions.

### 5.1 Career level

At different career levels, differing skills are required. To elaborate on that, we have prepared the following tables with three levels of experience in mind.

• Junior RSE: These are people who are in the earlier stages of their RSE career journey, but they should ideally have research experience of their own as well as the skills to contribute reliable and well-structured code to software projects.
• Senior RSE: They have gained experience, both concerning their software skills as well as in their research collaborations in potentially many different fields. They can set the standards in a software project.
• Principal RSE: Their actual job description varies a lot. These may be RSE team leaders based in a professional services type role, or they may be professors or research group leaders based in a more academic-focused role. They are often the people responsible for bringing in the funding that supports new and sustains existing projects. Generally speaking, they do not need to be actively involved in the day-to-day technical tasks, but they should be able to guide projects from both a technical and a research perspective while providing an inclusive working space.

Table 1, Table 2, and Table 3 elaborate on the required facets of the competencies in different roles. A story-like example of an individual through the hierarchies can be found in Section 5.4.2.

**Table 1.** Levels of technical skills expected per RSE career stage.

Competency	Junior RSE	Senior RSE	Principal RSE
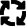 SWLC	Should be aware of the software life cycle.	Should know where in the life cycle their project is and which decisions are likely to lead to technical debt.	Should know how to manage and steer development/project resources accordingly. Should also have an understanding of the potential consequences of key project management decisions.
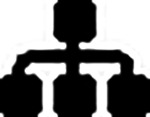 DOCBB	Should be able to write reusable building blocks.	Same as junior, but the quality should set the standard for the project, while following current best practices.	Should know the current best practices and point their team members and collaborators to the right resources.
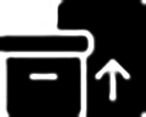 DIST	Should be able to use package distribution platforms.	Same as junior, but should also be familiar with current best practices for building and deploying packages.	Should ensure that their project is available via an up-to-date and secure distribution platform.
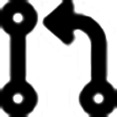 SWREPOS	Should seamlessly interact with the repository of their project.	Should be well-versed in the intricacies and best practices around working with a repository, and probably interact with repositories of multiple projects.	Should promote the use of repositories and be able to convey best practices of sharing and reviewing to junior and senior RSEs.
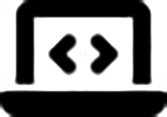 MOD	Should have a basic grasp of the part of the software they are responsible for in order to use basic tools such as a debugger.	Should understand the characteristics of large parts of the codebase considering a variety of the metrics.	Should have a detailed understanding of the software project as well as its aims and potential for impact, in order to effectively steer it.

**Table 2.** Levels of research skills expected per RSE career stage.

Competency	Junior RSE	Senior RSE	Principal RSE
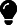 NEW	Should have some curiosity to fit into research teams.	Same as junior, but they should proactively propose directions in individual aspects of the project.	Should have research insights and a broad view of the research field to steer the project.
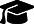 RC	Should be aware of the research life cycle.	Should know the position of the project in the research life cycle.	Should know what is necessary for the project to fit into its position in the research life cycle.
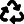 SRU	Should be aware of software reusability tools.	Should be able to search with software reusability tools.	Should be able to effectively search with SRU tools and to evaluate and perform the integration of a library into the project.
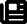 SP	Should be aware of available opportunities to publish software and understand the need to consider issues of intellectual property.	Should be able to correctly publish software in simple cases and to identify cases where professional legal advice is needed.	Same as senior, plus the ability to take the future publication of software into account when initiating and guiding larger software collaboration projects.
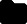 DOMREP	Should be able to interact with the domain repository.	Same as junior RSE.	Same as junior, and should know about how it fits into workflows surrounding these domain repositories.

**Table 3.** Levels of communication skills expected per RSE career stage.

Competency	Junior RSE	Senior RSE	Principal RSE
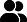 TEAM	Should be able to work in the team in order to effectively fulfil the given tasks. Should be able to learn from code review.	Should be able to break down tasks into more easily digestible sub-tasks and review or guide work undertaken by less-experienced team members.	Should be able to lead the team and set the respective direction.
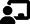 TEACH	Should be able to perform simple peer-to-peer on-boarding tasks.	Should be able to explain logical components and the general architecture to other RSEs.	Should be able to effectively communicate about all high-level parts of the project.
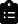 PM	Should be aware of the employed PM method.	Should be able to use and adapt the employed PM method.	Should be able to design and adapt the employed PM method.
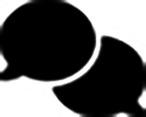 USERS	Should be able to communicate with both users and SEs on the project, on topics of the research and SE.	Same as junior RSE, and be able to interpret the feedback.	Same as senior, and should also be able to effectively take feedback into account when steering the project.

### 5.2 Helpful RSE skills for researchers in an academic career

In the previous section, we looked at the competency levels needed for RSE specialists. However, many of these competencies are important for domain researchers in academia as well, who do not specialise in RSE but nevertheless contribute to research software. Naturally, the ‘R’ competencies apply, and research in general is increasingly team based. Additionally, many researchers in fields from classical examples like numerical mathematics or theoretical physics to newer disciplines like digital humanities will spend time in their research on writing and developing software. Therefore, RSE focused training, e.g., in a master’s programme, is also beneficial for students in these fields resulting in a broader audience. This also means that students as well as researchers need to be given time to acquire those skills, e.g., to be able to attend training in RSE-relevant topics as part of their regular work or study.

This section outlines how the RSE competencies could be reflected at all academic levels. Again, this relates to domain studies and non-RSE positions in academia. It is important to note that this section does not reflect the current state of academic training and research institutions. Instead, it summarises the discussions with and between workshop participants at different levels of academic progression on what they would have liked to learn at an earlier stage or know before starting their current position. While individuals already work at implementing some of these changes and teaching these skills, it has not yet reached a systemic level.

The text is organised along the academic progression path (bachelor’s degree, master’s degree, PhD, Postdoc, Principal Investigator (PI)/Professor). Since each level is based on the previous levels, we presume that the skills and competencies at each level also encompass those of the previous levels. Due to the broad need throughout academic specialisations, the described levels serve as a baseline and certain fields will require higher SE skill levels as development is a large part of their actual research.

**Bachelor’s level** Students at the undergraduate level mostly consume science/knowledge. During their studies, they should also learn about the existence of digital tools and structures. Undergraduate students should be aware that RSEs exist and that software has different quality aspects ( 
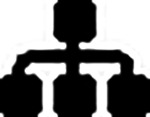
 DOCBB). They should be aware of domain specific tools ( 
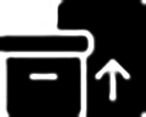
 DIST, 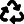 SRU) and where to find them ( 
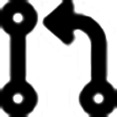
 SWREPOS, 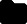 DOMREP). At this level, it may be sufficient to consider software as black boxes ( 
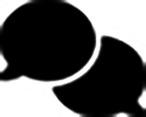
 USERS) although some training in data presentation would be very helpful and a good way to find out about programming ( 
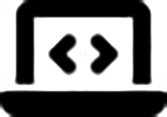
 MOD, 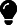 NEW). They should have a basic awareness of software licences, such as legal pitfalls and implications for good scientific practice ( 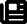 SP). They will be taught about the research cycle ( 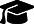 RC) and that researchers often work in groups ( 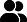 TEAM). During practicals, they will have an opportunity for peer learning ( 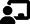 TEACH).

**Master’s level** A student at a master’s level can participate in science and should therefore be able to use “some” digital structures. A master’s student needs to be aware of relevant tools and data sets for their domain, where to find them and how to use them ( 
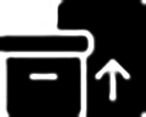
 DIST, 
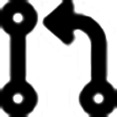
 SWREPOS, 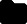 DOMREP). They should be able to process and present their data ( 
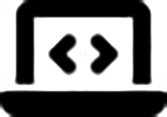
 MOD). They need to understand how their research depends on software ( 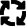 SWLC). Working on their master’s thesis allows them to understand the research cycle ( 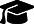 RC), practice project management ( 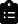 PM) and collaborate with other members of their research group ( 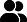 TEAM).

**PhD** PhD students perform independent research under guidance. They need to know relevant tools and structures. They should know where to find information about tools and where to find help using them ( 
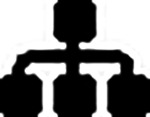
 DOCBB, 
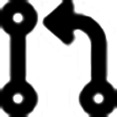
 SWREPOS). They should be able to use the tools ( 
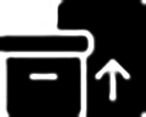
 DIST) and identify and report bugs ( 
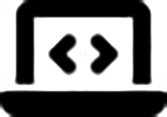
 MOD). They need to be aware that the user’s perspective is different from the developer’s perspective in order to be able to write good bug reports ( 
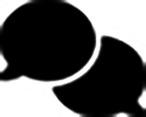
 USERS). They might produce new software ( 
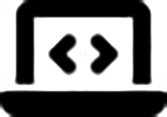
 MOD, 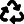 SRU), in which case they need to understand how to licence their code for publication ( 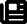 SP). PhD students need to be curious to be able to conduct their research. In order to be able to explore new tools ( 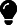 NEW) they must be able to evaluate research software ( 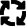 SWLC). They need to be able to interact with services ( 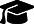 RC) and domain specific repositories ( 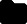 DOMREP). They should be able to supervise a student ( 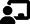 TEACH).

**Postdoc** Postdocs are independent researchers. Their role is similar to that of a PhD student, with a deepened focus on their research career. However, they are proficient users of all relevant tools, which makes them active contributors to their domain of research. They need to be aware of more advanced topics regarding intellectual property rights, such as patents ( 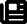 SP).

**PI/Professor** They are experts in their field and should be able to give proper guidance to their students on which digital tools are currently relevant. They should be aware of the skills of an RSE and when they might need one in their group. They should encourage their students to use relevant tools ( 
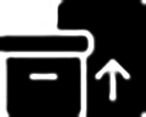
 DIST). They need to be able to judge the suitability of the software ( 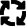 SWLC) and follow the interactions between relevant projects ( 
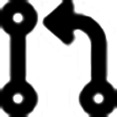
 SWREPOS). They should be able to advise their students on the legal aspects of software production and distribution ( 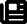 SP). They should be able to contribute meaningfully to the steering decisions of the software in their field ( 
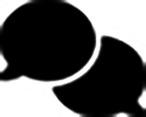
 USERS). They are able to guide students and prepare and deliver a full lecture course ( 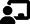 TEACH). They need to manage and lead their research group ( 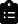 PM, 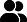 TEAM).

### 5.3 Project team structures

In Table 4, Table 5, and Table 6, we look at individual or team competencies and approaches to them, considering how these differ depending on whether an RSE is working alone on a software project, or whether they are working as part of a team of RSEs. We extend this to consider how things differ when an RSE or a group of RSEs is based locally within a research team or department, or when they are based in a dedicated, centralised RSE team. We also look at organisational aspects in the context of each of the considered competencies, since there are a variety of ways that organisations can contribute to and support them, complementing those proposed by Ref. 54. Some of them are brought to life in the example career path of Section 5.4.1. We first summarise the meaning of each of the columns in the tables:

• **Competency:** The code assigned to the competency being considered, as defined in Section 4, e.g. 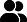 TEAM.
• **Individual RSE (Locally-based):** A single person working on software within a research project - for example a domain RSE with focus on their own specific research. Often time-constrained, may be self-taught.
• **Individual RSE (RSE team-based):** A single person working on research software - generally a professional RSE assigned to support another team’s software on their own, who however is connected to an RSE team.
• **Group of RSEs (Locally-based):** A group within a research group or team, working together on software to support or undertake a single research goal/project. Similarly to the individual RSE, they are often research-focused with RSE skills, often self-taught.
• **Group of RSEs (RSE team-based):** An RSE team working together on research software projects for a research group.
• **Organisation-level RSE support:** Describes how the defined competencies are recognised and represented at an organisational level and what the organisation can do to support the RSEs in the context of the different team structures. These can be read as policy/action recommendations.

These tables take the perspective of the expected skill set of each RSE or team of RSEs, similarly to personas in a user experience analysis. The current situation may differ.

**Table 4.** Levels of software eng. skills expected per team structure.

Competency	Working as an individual RSE		Working with a group of RSEs		Organisation-level support
	Locally-based	RSE-Team based	Locally-based	RSE-Team based	
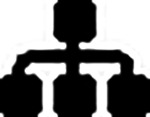 DOCBB	Focuses on supporting research. May not be very familiar with code quality and structure. Follows basic best practice guides.	Puts greater focus on reusability, documentation, and knowledge of best practices, but potentially lacks domain knowledge.	Has more opportunities to discuss and share ideas, but team members may be less aware of key practices.	Has stronger ingrained focus on team-base PM and development methodologies, resulting in higher quality, more reusable code.	Should offer training and other resources in core topics to support individual RSEs. Should have research software guidance/policies that provide advice.
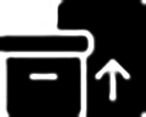 DIST	Does not emphasise code reusability and sharing/distribution.	Puts greater focus on reusability/sharing, but likely not as part of the project aims.	May want to develop reusable, shareable outputs for a specific case. Needs clear guidelines.	Focuses on quality and best practices. Reusability/packaging driven by project needs and spec.	Should provide policies on reusability/sharing. May be driven by requirements/policies, e.g., of institution or funding agency.
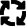 SWLC	Manages the complete life cycle, bus factor equal to 1.	The team supports parts of the software life cycle, but with low bus factor.	The team infrastructure and tooling supports the life cycle and sustainability.	The bus factor may still be low in parts of the code. Need to think about coherent life cycle management across the team - generally a key area of expertise for an RSE team.	Should support with training. Organisation may also provide site licences for, e.g., management tools.
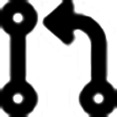 SWREPOS	Uses repositories for code management and demonstrating outputs, e.g., for supporting academic credit, but may be missing skills.	As locally-based, but professional RSEs are generally very experienced with use of repositories and their many features.	Uses repositories to collaborate inside the team. Can benefit from short courses on effective use.	Uses repositories extensively for project management, issue tracking, etc. in addition to code itself. May train others.	Should offer enterprise repository set ups, site licences etc. Also provide training for this vital research software development tooling.
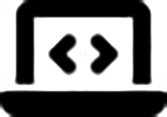 MOD	Needs full awareness of entire codebase to extend/maintain. If project taken on from another developer, there may be challenges in transferring the mental model.	As local, but more aware of need for future transition to other RSE(s), likely provides docs, issues, and other support from central services to support this. May only need to know parts of the code.	Internal team training ensures ability to build necessary mental model of codebase and to document it via text or tools for sustainability.	As local team, but likely more aware of tooling and practices in place within RSE team. Distributing work makes it only necessary for each developer to understand code related to their assigned tasks.	Should provide training and retain experience via coordinating and provide support for mentoring/community activities. Establishing RSE departments with specialists for certain aspects of software will improve overall turnaround times.

**Table 5.** Levels of research skills expected per team structure.

Competency	Working as an individual RSE		Working with a group of RSEs		Organisation-level support
	Locally-based	RSE-Team based	Locally-based	RSE-Team based	
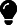 NEW	May struggle to learn new methods and skills due to split research focus between research goal and software project.	Gets support from the RSE team to explore new methods and skills, make relevant contacts and learn more about the domain.	Has increased interest in learning new methods and skills, but still prioritises domain research.	As team-based individual	Should reach out to relevant local groups to facilitate training and sharing of know-how on new technical processes and tooling.
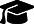 RC	Is familiar with the RC in their domain, especially when embedded in a research team.	Is familiar with the RC, although they may not have domain knowledge, which a group can provide.	Is familiar with the RC and can share knowledge within the team.	One or more members of the team are strongly aware of the RC.	Should provide extensive infrastructure to manage the RC, supporting researchers/RSEs.
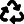 SRU	Has limited awareness of existing solutions and limited support regarding SRU.	Is familiar with software sharing and can discover tools and platforms.	As locally-based individual, but being part of a team can help to address this.	As team-based individual	Should run local environments to host software, catalogue software, and/or provide institution-level access to platforms that support this.
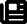 SP	Has limited knowledge and motivation regarding SP.	Applies practices, workflows, and policies established in the RSE team.	As locally-based RSE	As team-based RSE	Should raise awareness about software as a publishable scientific output, provide recommendations and checklists to support software publications, and have legal experts in place to offer advice on complex cases.
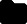 DOMREP	Domain researchers working on software are likely to be more familiar with the domain-specific solutions.	RSEs may need guidance from domain researchers around domain-specific repositories if they have a background in a different domain.	As locally-based individual	As team-based individual	Should host domain-specific repositories for areas that the organisation works extensively in, but this is likely to be handled at a research group level.

**Table 6.** Levels of communication skills expected per team structure.

Competency	Working as an individual RSE		Working with a group of RSEs		Organisation-level support
	Locally-based	RSE-Team based	Locally-based	RSE-Team based	
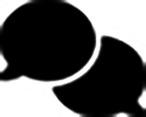 USERS	May have additional skills to safeguard potential future development and maintenance of the software for external users. Resourcing for future maintenance may be a challenge.	Has additional skills or can access support to safeguard potential future development and maintenance of the software for external users.	Needs to safeguard future development and maintenance of the software for external users, but may not have the skills or resources to support this.	Applies best practices to prepare the code for external users, while the team provides infrastructure and/or specialised RSEs for user support.	Should have institutions that are able to offer support with outreach and publicising outputs.
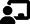 TEACH	May be independently involved in training activities.	May be able to support researchers with core technical skills.	Shares knowledge and skills within the group (peer support).	Supports teaching more widely, either through organised courses or ad hoc activities such as “code clinics”.	Should have programs for a diverse range of teaching/training activities, such as an RSE curriculum, as described in Section 5.4.1.
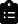 PM	Is organised enough to be able to transfer the codebase to future RSEs.	Follows the project management approach set by the team, or can suggest such PM approaches.	Has additional PM challenges, but may not have awareness of or experience with key PM skills, which can be acquired with low-key courses.	Team provides well-structured approaches and tooling to support management of projects.	Should offer training to support management of projects. May offer organisation-level tooling.
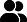 TEAM	When not developing code for themselves, they must be able to work effectively with researchers they are potentially developing code for.	Must be able to work effectively with their home RSE team, as well as with researchers they are potentially developing code for.	Must have strong team skills and knowledge to support team-based software development.	Must be able to work and collaborate effectively in an interdisciplinary team, use required tools and processes, infrastructure, etc.	Should offer support with team work and promote interdisciplinary interaction. Should facilitate team-building initiatives, also on a social level.

In the tables above, we have looked at how different competencies can be related to and handled by researchers and RSEs working in different environments within an organisation and how the organisations themselves can contribute. We recognise that this is a challenging area to gain a detailed view of and that this is still a significant generalisation. We talk about the “Research Software Engineer” as a single entity but as the field expands, we expect to see more roles and job titles emerging around the RSE concept, many of which fit under the wider umbrella of research technology professionals (RTPs). ^107^^,^^108^ Examples are different RSE-like computational roles of the EMBL-EBI BioExcel competency framework ^109^ (also Section 6.3.1), as is a range of different roles from King’s Digital Lab at King’s College London. ^83^

### 5.4 Designing a generalist RSE framework

**5.4.1 An example master’s programme for research software engineering**

The target audience for such a master’s programme are students holding a bachelor’s degree from a domain science, which we will call “home domain” in the following. There is explicitly no restriction on the candidates’ home domain: it may be from the STEM disciplines, life sciences, humanities or social sciences, and it can also change later in their career. Candidates with a bachelor’s degree in computer science are also explicitly included, although we acknowledge that their master’s programme should include adaptations to make their interaction effective with other domain scientists. In order to give the future RSE the necessary breadth, we expect this to be a four-semester curriculum.

The curriculum is formed from a combination of modules, some of which are core modules teaching essential skills that must be completed by all students. Other modules introduce more specialised concepts and skills. During the master’s programme, students should pick an RSE specialisation from the list in this paper and attend these additional modules to deepen their knowledge in that field.

Core modules are of course drawn from the three pillars of the RSE and can be categorised accordingly.

• Software/Technical skills:
  ○ Foundational module: Here we have an introduction to programming: Emphasising use cases over programming paradigms, students learn at least two languages: a language that facilitates prototyping and data processing (e.g., Python or R) and a language for designing complex, performance-critical systems (e.g., C/C++). This exposes them to computers in a hands-on fashion and is the foundation for ( 
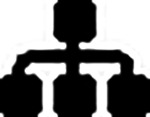
 DOCBB, 
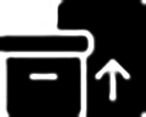
 DIST).
  ○ Computing environment module: Programming languages are not enough to work in a landscape of many interconnected software components; hence we require something like software craftsmanship, where tools such as the Unix shell, version control systems, build systems, documentation generators, package distribution platforms, and software discovery systems are taught to strengthen skills in ( 
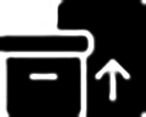
 DIST, 
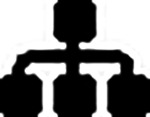
 DOCBB, 
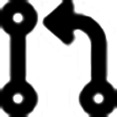
 SWREPOS, 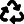 SRU).
  ○ Software engineering module: Here we develop foundational software engineering competencies (basic knowledge and skill regarding requirements engineering, software architecture and design, implementation, quality assurance, software evolution), again emphasising and strengthening ( 
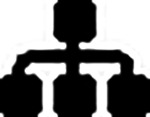
 DOCBB, 
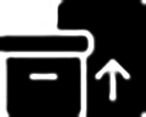
 DIST) on a more abstract level.
• Research skills:
  ○ Optional domain mastery module: Additional minor research courses, but students with a home-domain already have the research part well-covered. Courses here should be allowed to fall in any research field, and those outside of the own home-domain should be especially encouraged.
  ○ Research tools module: Here we teach tools used to distribute and publish software, as well as introducing students to domain specific data repositories, thereby gaining foundational knowledge in ( 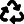 SRU, 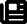 SP, 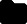 DOMREP).
  ○ Meta-research module: Here we teach people how research works. The research life cycle is introduced, as well as the data life cycle and the software life cycle are abstractly introduced.
• Communication skills:
  ○ Project management methods: Here we teach project management methods that are useful in science, such as agile ones ( 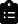 PM).
  ○ Communication skills module: Here we have courses focusing on interdisciplinary communication, interacting across cultures, communication in hierarchies, supporting end users effectively. These are all facets of the ( 
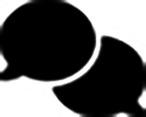
 USERS) skill.
  ○ Teaching module: This module covers topics to effectively design courses and teaching material for the various digital tools, thereby strengthening the ( 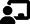 TEACH) skill.

Throughout the programme the values outlined in Section 3 are incorporated into the sessions to raise awareness of the codes of conduct and to put these values into ethical practice (see e.g. Ref. 13).

Given that RSE work also involves a lot of craftsmanship skills, hands-on practice is an integral part of the curriculum. At least two lab projects are required within the mandatory curriculum. These should be executed as a team and involve a question from a domain science. We recommend covering both the candidate’s home domain as well as a different one. Ideally, projects stem from collaborations with scientists within the institution and RSE students take the role of a consultant. This setup strengthens the ( 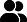 TEAM, 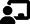 TEACH, 
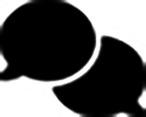
 USERS) skill and encourages also the ( 
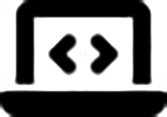
 MOD) skill through interaction.

To emphasise the exposure to domains outside their bachelor’s degree domain, we recommend that RSEs also support their non-home-domain project with introductory courses from this discipline. This schools their ability to quickly adapt their vocabulary and thinking to other disciplines and is an aspect of ( 
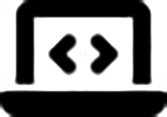
 MOD).

To align with the specialisations listed in this paper, example optional modules include topics on HPC engineering/parallel programming, numerical mathematics/scientific computing, web technologies, data stewardship, AI models/statistics, and community management/training.

The programme is finalised with a master’s thesis which should be dual-supervised by an RSE supervisor from an actual project, and a domain supervisor. The thesis should answer a relevant research question from the domain using computational methods, strengthening ( 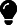 NEW). Software development is required, and the code is part of the gradable deliverables. The RSE supervisor ensures and grades the software craftsmanship aspects of the project. This setup ensures that we are grading the effectiveness of applying RSE skills in an actual research environment.

**5.4.2 An example of a possible career path**

**Setting the stage** Meet Kay, Kim’s ^2^ younger sister who currently studies researchology in a bachelor’s programme in the established domain of researchonomy at University of Orithena (UofO). We will follow Kay’s fictional career to illustrate how education, job-experience and a career in academic institutions could lead to become a successful RSE. In Kay’s world, some of the measures proposed in this paper have already been implemented.

**Bachelor’s degree** Through a program like Software Carpentry ^86^ or The Missing Semester, ^3^ Kay learns about using computational tools to support the sophisticated statistical analysis typical for researchology. She uses those tools to create and automate the steps of processing data and producing outcomes for her bachelor’s thesis (generating plots with matplotlib and even CI for automatic building) and takes pride in a fully open and reproducible bachelor’s thesis enabling her to graduate with honours from the faculty of researchonomy.

**Master’s degree** Kay ponders whether to continue with computational researchology, which her bachelor’s supervisor is responsible for, or enrol in a domain-agnostic RSE master’s programme. Researchers in computational researchology need to acquire a large part of the general RSE know-how presented in this paper and specialise in Quantum-Accelerated Bayesian Optimisation methods. However, Kay decides to go for the more generic route of a dedicated RSE programme because she wants to continue in academia, but does not like the idea of becoming stuck with one research topic. She also experienced the immediate satisfaction gained by helping colleagues from her research group with tricky technical problems, which makes her happier than the subdued sense of achievement from having a research paper accepted long after she had written it. For her, coding and sharing knowledge in the form of software is of similar importance to writing a paper focused mostly on the obtained results.

The domain-agnostic RSE Master programme consists of a core of RSE topics with various electives for specialisation, some of them domain-specific (e.g., chemistry) or topic-specific (e.g., cloud computing for research). Kay chooses digital archaeology and develops a pipeline for reconstructing 3D models from ground penetrating radar data, to simplify the process for archaeologists (reproducibility, big data, ML). The project management skills that are being taught as part of the core RSE curriculum really help her to not get lost in this project. Apart from working with the researchers in her archaeology group, she has to work with members of the central RSE department to help her with the pipelines. She also has to liaise with the central IT department to organise storage for the large data sets. Towards the end of the programme, she visits her first RSE conference where she sees a lot of notions ( 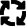 SWLC, 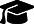 RC) in action that so far have been abstract in her master’s degree.

The exposure to the wider RSE community inspires her to invest additional time into her thesis to publish her software project under a licence approved by the Open Source Initiative and to write an accompanying article in the open source journal JOSS. ^53^ Inspired by the discussion with reviewers of her JOSS paper, and the citation metadata file that JOSS created automatically for her when her paper is published, Kay starts to think more about making her software FAIR. She reads up on the topic in a guide suggested to her, the Turing Way, ^91^ and creates metadata files that provide the citation metadata and general description for her software. She adds the files to her source code repository, and also adds an automated CI/CD pipeline that updates metadata and creates a new publication record in the Zenodo repository for each new release. Kay has now completed the RSE programme and has reached Junior RSE level.

**Junior RSE** Kay finds a position in the central RSE department at her university with a competitive IT salary. Although the contract is temporary, there is a good chance that it will lead to a permanent position. The university makes an effort to enable that since it is a member of “The Technician Commitment”, ^108^ an initiative to ensure recognition and career development of technicians, who face similar challenges to RSEs. Kay completes the Software Carpentry Instructor training and teaches basic research computing, while advising fellow students of her department on better programming ( 
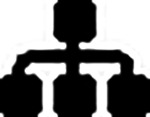
 DOCBB and 
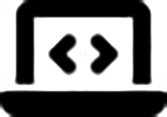
 MOD skill). She also runs a seminar in the RSE Master’s programme. She publishes a condensed version of that in JOSE. ^52^ During her teaching duties, she becomes aware of a new project in her department that requires a community manager RSE, and she gladly signs up to focus more on her communication skills. After three years, she takes an exciting opportunity to work in another university.

**Senior RSE** The new position involves taking responsibility for the RSE related aspects of a large inter-organisational project. With her new responsibilities comes a shift in the importance of various aspects of her work. Having this position in an inter-organisational project places far more emphasis on communication and organisation skills. She is spending time teaching people ( 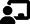 TEACH skill) to onboard them into the project. There is a lot of interaction with different stakeholders in the project like funders and user groups ( 
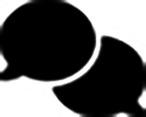
 USERS skill). To oversee the project, she uses an amalgamation of both agile and traditional project-management concepts and methods which she acquires on-the-job ( 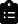 PM skills). Her work so far has already been heavy on ( 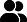 TEAM) skills, but now also the leadership aspect comes into play.

**RSE-focused principal investigator** The job experience as a leading RSE for a large project was the last requirement necessary to be awarded the title of a “Certified Research Software Professional” (CRSP) from an institutionalised centre of RSE education. The certificate confirms her track record of valuable software contributions and of teaching and mentoring people, as well as her capability to enable, foster and contribute to high-quality research in a leading position. It is recognised by various funding agencies, such as the DFG, and hence enables RSEs to act as a PI for RSE-focused grant applications. It is also recognised by many prestigious universities and opens many career options that are also typical for PhDs. Kay can now write her own grant proposals to effectively fund work of moving research software projects from prototypes to infrastructure.

## 6. RSE specialisations

What we have defined above is intended to be a set of skills that an RSE irrespective of domain, position, and experience should know about. There is a large variety of RSEs. They specialise in different areas, some of which we want to present below. Many of the specialisations may overlap, so the same RSE might for example work on data management and open science. We categorise them into those that can be viewed as a specialisation within RSE-specific topics, while other RSEs might expand their skill set and profession to areas that are not typical for an RSE.

### 6.1 Specialisations within the core RSE competencies

**Open science RSE** Open science and FAIRness of data and software are increasingly important topics in research, as exemplified by the demand of an increasing amount of research funding agencies requiring openness. Hence, an open science RSE is required to have a deeper knowledge of ( 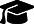 RC) and how to distribute software publicly ( 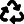 SRU, 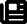 SP). Open Science RSEs can help researchers navigate the technical questions that come up when practising Open Science, such as “How do I make my code presentable?”, “How do I make my code citable?”, “What do I need to do to make my software FAIR?”, or “How do I sustainably work with an (international) team on a large code base?”. Like the Data-focused RSE, they have a deep understanding of research data management (RDM) topics.

**Project/community manager RSEs** When research software projects become larger, they need someone who manages processes and people. In practice, this concerns change management for code and documentation and community work to safeguard usability and adaptability, but also handling project governance and scalable decision-making processes. This gap can be filled by people who invest in the ( 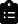 PM), ( 
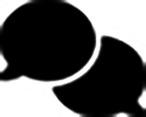
 USERS), and ( 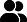 TEAM) skills, as exemplified in Section 5.4.2. Building a community around a research project is an important building block for sustainable software, ^80^ so these RSEs play an important role, even if they do not necessarily touch much of the code themselves.

**Teaching RSEs** RSEs interested in developing their ( 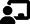 TEACH) skill can focus on teaching the next generation of researchers and/or RSEs and will play a vital role in improving the quality of research software. They need to have a good understanding of all RSE competencies relevant to their domain and additionally should have teaching experience and training in didactics and pedagogy.

**User interface/user experience designers for research software** Scientific software is a complex product that often needs to be refined in order to be usable even by other scientists. To facilitate this, there are people required that specialise in the ( 
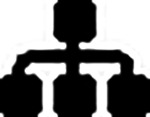
 DOCBB) and probably the ( 
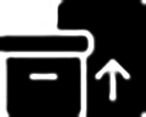
 DIST) competency with a focus on making end-user facing software really reusable and hence FAIR. This task is supported by strong ( 
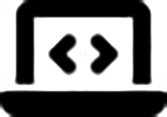
 MOD) skills to reason about the behaviour of potential users of the software.

### 6.2 Specialisations outside the core RSE competencies

**${DOMAIN}-RSE** While software is the common focus of all RSEs, there will be RSEs that have additionally specialised in the intricacies of one particular research domain, such as medical RSEs, digital humanities RSEs, or physics RSEs. This can often serve as a base domain for RSE specialisation as in Section 5.4.1.

**Data-focused RSE** Data-focused RSEs work at the flourishing intersection between data science and RSE. They are additionally skilled in cleaning data and/or running data analyses and can help researchers in setting up their analysis pipeline and/or RDM solutions. When the field requires research on sensitive data or information, e.g., patient information in medicine, this RSE should have knowledge about secure transfer methods and/or ways to anonymise the data. As part of RDM, this RSE profile is able to support all stages of the research data life cycle, ^70^ with synchronous data management processes. Those processes implement established best practices for planning and documenting of data acquisition in a data management plan (DMP), as well as for management, storage, and preservation of data, and publication and sharing of data in repositories according to the FAIR principles. ^98^

**Research infrastructure RSE** This RSE has a special interest in SysOps and system administration and sets up IT infrastructures for and with researchers. Therefore, this specialisation on the one hand requires a deep knowledge of physical computer and network hardware and on the other hand knowledge about setup and configuration of particular server software, e.g., setup of virtual machines on hypervisors or the planning and setup of compute server clusters for special purposes, e.g., ML. As an interface between the researchers and the infrastructure, they take care of user management, access permissions, and configuration of required services.

**HPC-RSE** RSEs with a focus on HPC have specialist knowledge about programming models that can be used to efficiently undertake large-scale computations on parallel computing clusters. They may have knowledge of (automatic) code optimisation tools and methods and will understand how to write code that is optimised for different types of computing platforms, leveraging various efficiency related features of the target hardware. They are familiar with HPC-specific package managers and can build dependencies from sources. They also understand the process of interacting with job scheduling systems that are often used on HPC clusters to manage the queuing and running of computational tasks. HPC-focused RSEs may be involved with managing HPC infrastructure at the hardware or software level (or both) and understand how to calculate the environmental impact of large-scale computations. Their knowledge of how to run HPC jobs and write successful HPC access proposals can be vitally important to researchers wanting to make use of HPC infrastructure.

**ML-RSE** The development of research software based on ML requires additional specialised theoretical background and experienced handling of appropriate software in order to produce meaningful results. This involves knowledge about data analysis and feature engineering, metrics that are involved in ML, ML algorithm selection and cross validation, and knowledge in mathematical optimisation methods and statistics. Here, we use ML in a broad sense of machine-based learning including deep learning, reinforcement learning, neuro-symbolic learning and similar.

ML-RSEs analyse and check the suitability of an algorithm. They check if it fulfils the needs of a certain task and they play a central role in deciding on and selecting ML libraries for a given task. The increasing usage of ML in numerous scientific areas with social impact involves an emphasised awareness and consideration of possible influences and biases. At the intersection of data science ^32^ and data-focused RSEs, the complex way of solving problems utilising ML calls for this separate specialisation.

**Legacy RSEs** Research software may have evolved over generations of researchers without change management or governance processes, while software “ecosystems” (e.g., programming languages, frameworks, operating systems) constantly evolve. This may lead to the emergence of legacy code that is still actively used. To safeguard continued usability and adoption, these RSEs have experience in working with code written in language standards and on software stacks considered deprecated by their communities. Adaption of existing, large-scale codebases to evolving dependencies ( 
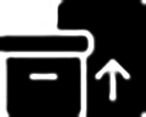
 DIST) or changing hardware (HPC; see the HPC-RSE specialisation) may require mastery in refactoring techniques and in the usage of specialised code transformation tools.

**Web-development RSE** This RSE is skilled in the development of web applications and/or mobile apps. They have expertise in one or more of frontend development, backend development and the design or implementation of APIs, for example to support research data portals or big research projects. Since a lot of web services for research may be accessible to a large audience or even to the public, this RSE is also familiar with aspects relating to cybersecurity, usability and accessibility. Not only do they need to balance these concerns while adhering to their values from Section 3, but they also need to efficiently communicate the decisions made to stakeholders.

**Legal-RSE** RSEs are often the go-to person for questions about software licensing, in particular when mixing software components that use different licences. But with the rising requirements from legislation, we foresee the need for RSEs that still have a background in RSE but extend it with a knowledge of legal processes that cover corner cases and go beyond applying Best Practice guides. These requirements may arise in the area of publication of research software, as this also requires knowledge about particular laws or regulatory frameworks concerning data protection, like the General Data Protection Regulation (GDPR) within the European Union (EU). ^87^ Another area are legal aspects of cybersecurity and export control in science and research (see Ref. 110 for Germany). Legal-RSEs focus on facilitating the achievement of technically feasible solutions, while adhering to regulatory mandates. They are able to communicate and collaborate effectively with lawyers.

### 6.3 Existing frameworks for specialised RSE roles

**6.3.1 Bioinformatics skills and certification**

Bioinformatics is another field that actively works on developing skill trees. The Bioinformatics Core Competencies, ^66, 95, 96^ the BioExcel competency framework, ^64^ the PerMedCoE competency framework, ^61^ the Research Data Management and Data Stewardship competence framework ^22^ and the ELIXIR Data Stewardship Competency Framework for Life Sciences ^79^ are examples of grassroots efforts aiming at defining the set of skills of various bioinformatics specialities, one of them as a taxonomy. ^66^ These frameworks eventually converged into the EMBL-EBI Competency Hub, ^25, 62^ where typical RSE and bioinformatician profiles at different levels of seniority can be queried (e.g., Junior RSE, Senior Computational Chemist) and compared against one another (e.g., Junior vs. Senior RSE).

Competencies can be divided into more fine-grained building blocks: knowledge, skills and abilities (KSAs). They can be organised in a taxonomy, and are also transferable, i.e. a KSA can be a prerequisite to multiple competencies. The Mastery Rubric for Bioinformatics ^92^ and the ELIXIR Data Stewardship Competency Framework for Life Sciences ^79^ are examples of KSA frameworks for bioinformatics curricula.

The Curriculum Task Force of the International Society for Computational Biology (ISCB) curates a database of degrees and certificates in bioinformatics. ^49, 66^ The database includes bachelor’s and master’s degree programs and specialisations, PhD programs, and certificates from graduate schools.

BioExcel has research competencies that combine some of our research competencies and some notions from the communication skills. Their computing competencies roughly map to our software skills. Here, we find competencies such as “package and distribute software”, which maps to our ( 
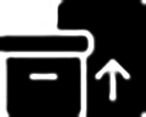
 DIST) competencies, and “comply with licensing policy”, which would in our framework be part of ( 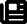 SP) in the research competencies. In addition, they have a dedicated parallel computing competency section, thereby shifting the emphasis of the knowledge of their computational tools towards the HPC-RSE specialisation in our framework. Career profiles, such as the computational chemist, bring additional domain specific knowledge; we would classify those as a mixture of ${DOMAIN}-RSE and HPC-RSE. It is noteworthy, however, that the BioExcel framework puts very little emphasis on communication skills, which are often involved in RSE-related tasks.

**6.3.2 HPC skills and certification**

As an area that generally requires a range of advanced skills, HPC is one field where there is ongoing work to identify relevant sets of skills for HPC practitioners and potential paths to develop these skills. The HPC Certification Forum ^89^ has developed a competence standard for HPC that defines a range of skills and how they are related in the context of a skill tree. ^55, 56^ This competence standard is currently being built upon by the CASTIEL 2 ^29^ project in collaboration with initiatives funded by the European High-Performance Computing Joint Undertaking (EuroHPC JU) to create a framework for HPC certification. ^39^ While this framework focuses mostly on skills specific to HPC, there are a couple of similarities to the framework proposed in this paper. The “SD: Software Development” skill set is very similar to the SE skills discussed in Section 4, describing a wide range of such skills. This skill set contains Programming Best Practices (SD2), Software Configuration Management (SD3), Software Quality (SD5), Software Design and Software Architecture (SD6), and explicit mention of documentation (SD7, see our 
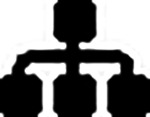
 DOCBB). Besides the Software Concepts for HPC (SD1), which mainly concerns HPC-focused RSEs, most of the skills contained in the SD2-SD7 categories apply to all RSEs. A significant difference compared to the framework proposed in this paper is the absence of skills related to research or communication. Noteworthy is already now the level of detail in their skill tree which is more similar to Section 5.4.1.

Also looking at pathways and how different skills are related, the UNIVERSE-HPC project, ^93^ funded under the UK’s ExCALIBUR research programme, ^30^ is looking to understand and develop training pathways to support the development of specialist skills in the HPC and exascale domains. The project is gathering open source training materials to develop curricula that support the training pathways that are underpinned by high-quality training materials.

## 7. Future work

This list and description of competencies is a first step to finding common ground around which to structure curricula, institutions, and teachers in this framework. Applications of these competencies in an individual’s career can be found in Section 5.4.1. Opportunities for sustainable funding is a concern that is often ^12^^,^^15^^,^^36^^,^^68^ raised by RSEs. An omission that we found and that we would like to highlight in order to spark a community discussion is that RSEs that choose explicitly a science-supporting role outside of research will not be eligible for funding under the statutes of many funding organisations that require at least a PhD.

To alleviate this and to give RSEs in leadership positions a means to become eligible for funding themselves, since completion of scientific training is often a requirement, ^34^ we see two possible parts of a solution. One is to allow for doctorates primarily based on software contributions to the scientific community. Secondly, we propose the introduction of new, standardised certificates like those of Section 5.4.2, and to officially accept them as PhD-equivalent concerning eligibility to be a PI. Beyond this discussion, a diverse set of publications on the topic of RSE teaching is already in the making.

Within this set, we will work next on how to institutionalise education. In that publication, we will detail how we organise our institutions and what qualifications our teachers need to have in order to effectively communicate our values. We will put forward ideas on how to build up bachelor’s and master’s programmes, of which a glimpse can already be found in Section 5.4.1. We will show how we intend to provide the necessary continuous education for RSEs after graduation, and we will connect that with the integration of RSEs into a mesh of community networks aimed at supporting research, while providing them with an inclusive social network that further facilitates lifelong learning. That publication will again intentionally be free of regional specifics, to also serve as a blueprint that other national RSE societies can build upon.

Online resources for courses are another important building block. This is the general intention of the learn-and-teach project. ^90^ Surveying and curating of existing resources is not carried out as a traditional publication, but it is made available as a continuously-evolving online resource at. ^90^

And finally, we plan to formulate a call to action, building on the previously mentioned publication on the necessary institutions, that spells out everything that is required to best support the continuous need for young RSEs to support digital science specifically in Germany.

## 8. Conclusion

This paper started from a community workshop at deRSE23 in Paderborn where people working in RSE related fields got together to figure out structures and ideas for educating newcomers to this field. One outcome of this diverse gathering is that RSEs from differing fields gather around similar core concepts, At the same time they share a vision of how to renew scientific research practice making extensive use of digital tools. In this publication, we have tried to formalise these concepts. We have formulated a set of values that guide our actions in society, manifestly making RSEs part of the scientific community that shares the ideals of good scientific practice. At the same time, being close to software engineers, we cherish that we have to take responsibility for our tools. We listed core competencies that have been intentionally formulated abstractly without referencing any particular information-processing device. As expected, we have drawn equally upon notions from SE and other research fields, but found that we likewise require teamwork capabilities. We detailed these competencies in various dimensions and found that a different amount is required in different positions and scientific domains. Using this, we proposed recommendations for organisations to foster the development of these competencies.

The gathered values and competencies form a common denominator that unifies RSEs and enables them to identify with this domain, in the knowledge that it is already or will soon become critically important for many areas of science. These competencies at the intersection of research and SE, coupled with a firm belief in team processes, make RSEs sought after on the job market and their values make them responsible members of a digital society. The result is a qualification profile which is highly attractive for young people.

At an institutional level, research performing organisations have a growing interest in fostering RSE training to support the use of FAIR data and FAIR software in the academic world, a direction determined by new incentives created by scientific journals and librarians. How we update existing institutions and set up new ones that provide this education will be the topic of a follow-up paper.

### Contribution details

Heidi Seibold came up with the original idea for the deRSE23 workshop in Paderborn. Heidi Seibold, Jeremy Cohen, Florian Goth, Renato Alves, Jan Philipp Thiele, and Samantha Wittke organised the deRSE23 workshop. We thank all the participants of this community workshop! Toby Hodges conceptualised and organised the un-deRSE23 workshop together with Jan Philipp Thiele and Florian Goth. We also thank all the participants of this follow-up community workshop! Jeremy Cohen, Gerasimos Chourdakis, Magnus Hagdorn, Jean-Noël Grad, Jan Philipp Thiele, and Matthias Braun organised the deRSE24 workshop in Würzburg. We are also grateful to the participants of this third community workshop! Heidi Seibold, Jeremy Cohen, Florian Goth, Renato Alves, Jan Philipp Thiele, Jan Linxweiler, Jean-Noël Grad, and Samantha Wittke contributed the initial draft. Florian Goth supervised the project and did the project administration. Jean-Noël Grad designed and implemented the software tooling for the collaborative writing of this manuscript on GitHub. Everybody contributed to the final review and editing.

The CRediT system ^11^ is far too generic to adequately describe the contributions of everybody in various workshops, spread over a two year period. While everybody contributed to the discussion formulating and refining the ideas, and to collaboratively writing and/or reviewing and editing the entirety of the script, some parts merit special mention. Renato Alves quickly jumped in to host the first deRSE23 workshop to take over from a sick organiser. Matthias Braun contributed early versions of the specialisations and also contributed to the survey. Leyla Jael Castro contributed to the initial draft of the example career path, and provided helpful insights in discussions on metadata. Gerasimos Chourdakis’ contributions to the paper are numerous (extensively editing large parts of the initial draft), but he especially wrote first drafts for clarifying the relationship of the RSE competencies to the SE competencies. He also designed the “Learning and teaching RSE” website. ^90^ Simon Christ helped with typesetting and contributed the competencies’ symbols and the spell-checker script. Jeremy Cohen drafted the initial introduction and contributed the tables for RSEs in centralised RSE departments. Stephan Druskat contributed parts on proper software citation and publication and sharpened various RSE specialisations. Fredo Erxleben contributed to early discussions of the paper and added the contributions by the Helmholtz Association. Jean-Noël Grad contributed initial drafts for the ELIXIR framework and the section on the work of the HPC certification forum as well as numerous other contributions to the BibTeX infrastructure and the GitHub actions. Magnus Hagdorn drafted and supervised the ethics and values section for an RSE and made sure that these values are reflected in the competencies of RSEs. Toby Hodges contributed parts on the Carpentries, and helped steer the curriculum discussion. Guido Juckeland contributed experiences from his first RSE course for students. Dominic Kempf drafted the first version of the example curriculum. Anna-Lena Lamprecht helped with proper wording, especially with awareness about established SE terminology, that was misused earlier. Jan Linxweiler drafted various RSE specialisations and made sure that clean coding techniques got their due recognition. Frank Löffler rewrote numerous parts to be actually legible, and helped with preparation for the final steps of a de-RSE position paper. Michele Martone wrote the first draft of the environmental sustainability section. Moritz Schwarzmeier drafted the categorisation of the specialisations. Heidi Seibold contributed the idea and started everything. Jan Philipp Thiele drafted initial parts of the technical pillar of the RSE competencies, and represented the project on numerous discussions. Harald von Waldow contributed to initial drafts of the Masters program and contributed his knowledge to the explainability of computer simulations. Samantha Wittke contributed the parts on CodeRefinery and how to reach out to new RSEs. Florian Goth has the pleasure of being grateful to all collaborators in this project for contributing their time and knowledge into this project!

## Ethics and consent

Ethical approval and consent were not required.

## Glossary

**bus factor** vulnerability of a project to losing key and irreplaceable team members - a bus factor of 1 means that a single such team member vanishing would already stall the project’s advancement.

**C** general-purpose compiled programming language.

**C++** general-purpose compiled programming language.

**design pattern** general and reusable solution to solve a SE problem (often a best practice, or a “recipe”).

**Forge** A platform that integrates software repositories and other communication or project management tools.

**GitHub** online software repository hosting and collaboration platform.

**GitLab** online software repository hosting and collaboration platform.

**Python** general-purpose scripting language.

**R** general-purpose scripting language.

**software publication** the practice of long-term archiving software artifacts with software metadata under a permanent identifier.

**static analysis** automated procedure to detect software bugs in source code without executing the code.

**SysOp** system administrator in charge of a computing infrastructure.

## Skill codes


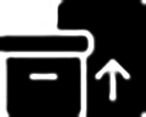
 **DIST** Building distributable software. Section 4.1.4


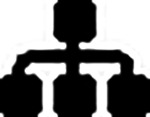
 **DOCBB** Creating documented code building blocks. Section 4.1.3


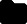
 **DOMREP** Using domain repositories/directories. Section 4.2.5


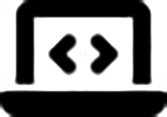
 **MOD** Software behaviour awareness and analysis. Section 4.1.6


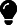
 **NEW** Conducting and leading research. Section 4.2.1


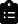
 **PM** Project management. Section 4.3.3


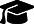
 **RC** Understanding the research cycle. Section 4.2.2


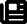
 **SP** Software publication and citation. Section 4.2.4


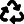
 **SRU** Software re-use. Section 4.2.3


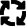
 **SWLC** Adapting to the software life cycle. Section 4.1.2


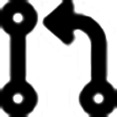
 **SWREPOS** Use software repositories. Section 4.1.5


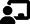
 **TEACH** Teaching. Section 4.3.2


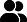
 **TEAM** Working in a team. Section 4.3.1


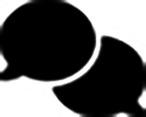
 **USERS** Interaction with users and other stakeholders. Section 4.3.4

## Acronyms

**CD** continuous delivery.

**CI** continuous integration.

**CI/CD** continuous integration and continuous delivery.

**DFG** German Research Foundation (Deutsche Forschungsgemeinschaft).

**DMP** data management plan.

**EMBL-EBI** European Molecular Biology Laboratory - European Bioinformatics Institute.

**ENCCS** EuroCC National Competence Center Sweden.

**EU** European Union.

**EuroHPC JU** European High-Performance Computing Joint Undertaking.

**FAIR** Findability, Accessibility, Interoperability and Reusability.

**GDPR** General Data Protection Regulation.

**GPL** GNU General Public License.

**GREENER** Governance, Responsibility, Estimation, Energy and embodied impacts, New collaborations, Education and Research.

**HIFIS** Helmholtz Federated IT Services.

**HPC** High-Performance Computing.

**IDE** integrated development environment.

**ISCB** International Society for Computational Biology.

**IT** information technology.

**LLM** large language model.

**MIT** Massachusetts Institute of Technology.

**ML** machine learning.

**NFDI** National Research Data Infrastructure (Nationale Forschungsdateninfrastruktur).

**PI** Principal Investigator.

**PRACE** Partnership for Advanced Computing in Europe.

**RDM** research data management.

**SE** software engineering.

**STEM** science, technology, engineering and mathematics.

**TDD** test-driven development.

**UK** United Kingdom.

**UML** Unified Modelling Language.

**UNESCO** United Nations Educational, Scientific and Cultural Organization.

**UNIVERSE-HPC** Understanding and Nurturing an Integrated Vision for Education in RSE and HPC.

## Data Availability

No data associated with this article.
