# Supplementary material for: Foundational Competencies and Responsibilities of a Research Software Engineer: Current State and Suggestions for Future Directions
Source: F1000Res. 2025 Sep 8;13:1429. Originally published 2024 Nov 26. [Version 2] doi: 10.12688/f1000research.157778.2 (PMC12426816; doi:10.12688/f1000research.157778.2)
Supplement: Peer review history

# Reviewer report: Reviewer response for version 1

**DOI:** 10.5256/f1000research.173278.r345501

**Published:** 2024-12-26

**Authors:** Uwe Schmitt

**Affiliations:** 1 ETH Zurich, Zürich, Zurich, Switzerland

# Summary

 The paper addresses many aspects of working as an RSE in different roles and the skills required or recommended to work effectively and to a high standard in this area.  As outlined, RSEs work in a wide variety of roles and as such the inclusion of many contributors helps to ensure that many different aspects are covered and considered.

 In addition, the authors outline future scenarios on how the current situation can be systematically shaped and improved. The paper is well structured and readable and makes a significant contribution to the understanding and future development of different RSE roles, required skills and training and career paths. The authors also outline future publications based on this article.

 # Minor corrections and suggestions.

 I think the title of the publication only describes part of the content and does not include the proposed suggestions and scenarios for the future of RSE skills development and support. A different title, along the lines of "Foundational Competencies and Responsibilities of a Research Software Engineer: Current State and Suggestions for Future Directions" might better describe the content of the article. 

 ## Section 3 (Values)

 This sentence appears to be corrupted due to editing:

 “Central to that code is the RSE’s obligation to In addition to the

 values for good scientific practice commit to the health, safety and welfare of the public and act in the interest of society, their employer and their clients.”

 “RSEs also adhere to the SE Code of Ethics”: As much as I agree with the proposed values, I would prefer the subjunctive/conjunctive form here, as used in other parts of the document. The teaching of these values could be strengthened in the later section proposing bachelor and master curricula.

 ## Page 14 bottom:

 I recommend to separate the following two paragraphs:

 RSEs are also mentoring colleagues (see also Section 3.1.2). This necessitates giving good advice that fits to a project’s

 stage in its life cycle, thereby requiring knowledge of ( SWLC), and its context in its research domain and thus ( RC).

 **END OF PARAGRAPH HERE**

 Research software can often start out as a tool to answer a personal research question, becoming more important

 when other researchers start to rely on it. At the other end of the scale, research software can sometimes underpin key

 processes that deal with critical questions such as weather forecasting or medical diagnosis.

 ## Table 4

 The row “SLWC” mentions “bus factors” several times and may need  explanation, or should be added to the glossary.

 ## 5.4.1

 Indentation of the bullet points after “Research skills” seems to be broken.

Is the topic of the opinion article discussed accurately in the context of the current literature?

Yes

Are arguments sufficiently supported by evidence from the published literature?

Yes

Are all factual statements correct and adequately supported by citations?

Yes

Are the conclusions drawn balanced and justified on the basis of the presented arguments?

Yes

Reviewer Expertise:

Senior RSE in different scientific domains.

I confirm that I have read this submission and believe that I have an appropriate level of expertise to confirm that it is of an acceptable scientific standard.

# Response

**Published:** 2025-07-18

**Authors:** Florian Goth

**Affiliations:** Julius-Maximilians-Universitat Wurzburg, Würzburg, Bavaria, Germany

Dear reviewer,

 we thank you for your constructive feedback and have used it for an improved version, that will be public here shortly. Here is how we adapted the manuscript in detail:

 ## Reviewer 2: Section 3(Values)

 > This sentence appears to be corrupted due to editing:

 > “Central to that code is the RSE’s obligation to In addition to the

 > values for good scientific practice commit to the health, safety and welfare of the public and act in the interest of society, their employer and their clients.”

 > “RSEs also adhere to the SE Code of Ethics”: As much as I agree with the proposed values, I would prefer the subjunctive/conjunctive form here, as used in other parts of the document. The teaching of these values could be strengthened in the later section proposing bachelor and master curricula.

 - corrupted sentence is fixed in markdown version of the paper

 - changed "RSEs also adhere to the SE Code of Ethics..." to "RSEs also need to adhere to..." to indicate that this is an aspiration

 - good point to refer back to values from the example curriculum. We have expanded the example curriculum accordingly.

 ## Reviewer 2 (GH: #395) Reviewer 2 Feedback: page 14 bottom

 > I recommend to separate the following two paragraphs:

 > RSEs are also mentoring colleagues (see also Section 3.1.2). This necessitates giving good advice that fits to a project’s

 > stage in its life cycle, thereby requiring knowledge of ( SWLC), and its context in its research domain and thus ( RC).

 > END OF PARAGRAPH HERE

 > Research software can often start out as a tool to answer a personal research question, becoming more important

 > when other researchers start to rely on it. At the other end of the scale, research software can sometimes underpin key

 > processes that deal with critical questions such as weather forecasting or medical diagnosis.

 - We added a line break at the indicated position.

 ## Reviewer 2:

 > Indentation of the bullet points after “Research skills” seems to be broken.

 Thanks for the feedback, We fixed the indentation in 5.4.1. While at it, we also regenerated the table of contents for F1000.

 ## Reviewer 2:

 > The row “SLWC” mentions “bus factors” several times and may need explanation, or should be added to the glossary.

 We checked, the bus factor is referenced in the glossary. We have used the opportunity, to include an html link to the glossary here.

# Reviewer report: Reviewer response for version 1

**DOI:** 10.5256/f1000research.173278.r345505

**Published:** 2024-12-17

**Authors:** Miranda Mundt

**Affiliations:** 1 Sandia National Laboratories, New Mexico, USA

# Paper Review - Foundational Competencies and Responsibilities of an RSE

 ## Summary

 This paper presents an idyllic image of what RSE education and career paths could be. It presents a detailed (but high-level) overview of competencies and provides example specializations. It is unclear the full intended scope (i.e., is this meant to be an emphasis on RSEs only in academia? Only in Europe? All RSEs in all roles worldwide?) Overall, this is clearly a paper that is well-considered, well-formatted, and a labor of love.

 ## Specific Feedback

 - Scope: How large is the scope of this paper? Is it meant to encompass RSEs around the globe or focused primarily on European RSEs? Also is it meant to be aspirational (e.g., all RSEs should have these) or does it represent the current state of RSEs (e.g., most RSEs already have these competencies)?

 - Section 2 (Related Work):

   - Related to scope question: If this is intended to be more widespread, I recommend including references to INTERSECT (https://intersect-training.org), BSSw (https://bssw.io), and IDEAS-productivity (https://ideas-productivity.org)

 - Section 3 (Values)

   - I'd push back on the claim that RSEs adhere to the SE Code of Ethics purely because, as stated earlier in the paper, a lot of RSEs don't have classical training and wouldn't even necessarily know that those ethics exist. Do they probably adhere to them accidentally? Yes. But I'd suspect the average RSE couldn't reliably list anything in the SE Code of Ethics.

   - First paragraph, something is missing here / incomplete sentence/thought: "Central to that code is the RSE's obligation to In addition to the values..."

   - I had a bit of a hard time following the computer-based modeling paragraphs / understanding why a discussion of them was happening until the second to last sentence of the paragraph that starts with "The relationship between initial state..."

 - Section 3.1 (Challenges)

   - I notice that "funding methods" is missing from these challenges; there has been a lot of discussion about unreliable funding methods for software and, as a result, RSEs (e.g., https://ieeexplore.ieee.org/stamp/stamp.jsp?arnumber=6886129, https://ieeexplore.ieee.org/stamp/stamp.jsp?arnumber=9470770, https://pure.manchester.ac.uk/ws/portalfiles/portal/54140648/StateOfTheNationReport2017.pdf, https://ieeexplore.ieee.org/stamp/stamp.jsp?arnumber=10071971)

   - There is also a lack of "RSEs being integrated into existing teams and having to fight that team's culture". Maybe this is too niche to bring up, but it is a challenge that has been spoken about amongst RSE circles (e.g., "How can I get an existing team to change their processes when they are so stuck in their ways?")

   - Section 3.1.1 - this almost might be better if phrased as "Data Security" because that's kind of the crux of the paragraph

   - Section 3.1.2 - I agree with all of the points here *but* it's realistically not just up to RSEs to embrace and shape diversity. What does it mean for them to "mitigate them whenever they have the chance to do so"? Examples might be helpful.

 - Section 4 - can you include a footnote to any digital information on the Paderborn workshop?

 - Section 4.1.2 - there are multiple research software maturity models/frameworks that have been proposed that adapt SWLC. See, for example, M. R. Mundt, W. Burgess and D. M. Vigil, "A Tiered Approach to Scientific Software Quality Practices," in Proceedings of the 2022 Improving Scientific Software Conference (No. NCAR/TN-574+PROC). doi:10.5065/98kd-b491

 - Section 4.1.5 - I recommend adding a note here for "as applicable," e.g., some RSEs at some institutions may not be able to share code publicly because of security or institutional requirements (though they should still use whatever they can for version controlling)

 - Section 4.2.4 - Similar note to 4.1.5; there should be something about "adhering to institutional policies" as well

 - Section 4.4 (Tasks and Responsibilities)

   - When talking about RSE communities, it might be worth it to reference some of them, e.g., UK-RSE / Soc RSE, deRSE, US-RSE, RSE-AUNZ, RSE Asia, RSSE Africa, etc.

 - Section 5.2 (Helpful RSE skills in academic career)

   - I disagree with some of the claims here (e.g., license discussion for Bachelor's degree seems like too much). Generally, though, this section is nice. However...

   - Not all RSEs have PhDs. Most of them do, yes, but many RSEs who do NOT have PhDs are still wildly successful without having gained the proposed skills as PhD students. How do you address that scenario (i.e., where an RSE stops after a Bachelors, Masters, etc.?)

 - Section 5.3 (Project team structures) - there is a gap here: "The single RSE on a team of not-RSEs who does not have a central RSE team connection." These do exist (sadly).

 - Section 5.4.1 - formatting weirdness in the Research Skills sub-list (pg 22)

 - Section 6 - This section is my favorite. I love the examples of different specializations because it allows a lot of readers to "see" themselves in this paper.

Is the topic of the opinion article discussed accurately in the context of the current literature?

Partly

Are arguments sufficiently supported by evidence from the published literature?

Yes

Are all factual statements correct and adequately supported by citations?

Yes

Are the conclusions drawn balanced and justified on the basis of the presented arguments?

Yes

Reviewer Expertise:

Research software engineering with an emphasis on testing, reproducibility, and diversity; FAIR(ER) principles (E = Equitable, R = Realistic)

I confirm that I have read this submission and believe that I have an appropriate level of expertise to confirm that it is of an acceptable scientific standard.

# Response

**Published:** 2025-07-18

**Authors:** Florian Goth

**Affiliations:** Julius-Maximilians-Universitat Wurzburg, Würzburg, Bavaria, Germany

Dear reviewer,

 We thank you for your constructive comments, and a future update will include changes to reflect your feedback. Here is what we did in detail:

 ## Reviewer 1: Scope

 > Scope: How large is the scope of this paper?

 > Is it meant to encompass RSEs around the globe or focused primarily on European RSEs?

 > Also is it meant to be aspirational (e.g., all RSEs should have these)

 > or does it represent the current state of RSEs (e.g., most RSEs already have these competencies)?

 We acknowledge that the scope and the perspective might be confusing in parts.

 We have extended the abstract and the introduction to clarify that

 the foundational competencies are based on what is often encountered in Germany and beyond,

 while aiming to be guidelines for aspiring RSEs.

 We have also clarified that our recommendations draw from experiences in Germany, Europe, and the US,

 but are not specific to a particular region.

 A subtitle was added to better reflect the scope and aims of the paper.

 ## Reviewer 1: Related work

 > - Section 2 (Related Work):

 >    - Related to scope question: If this is intended to be more widespread, I recommend including references to

 >      INTERSECT (https://intersect-training.org),

 >      BSSw (https://bssw.io), and

 >      IDEAS-productivity (https://ideas-productivity.org)

 We have now added these examples from the USA, which we previously missed. Thank you for reporting these blind spots!

 ## Reviewer 1: Section 3 (Values)

 > I'd push back on the claim that RSEs adhere to the SE Code of Ethics purely because, as stated earlier in the paper, a lot of RSEs don't have classical training and wouldn't even necessarily know that those ethics exist. Do they probably adhere to them accidentally? Yes. But I'd suspect the average RSE couldn't reliably list anything in the SE Code of Ethics.

 > First paragraph, something is missing here / incomplete sentence/thought: "Central to that code is the RSE's obligation to In addition to the values..."

 > I had a bit of a hard time following the computer-based modeling paragraphs / understanding why a discussion of them was happening until the second to last sentence of the paragraph that starts with "The relationship between initial state..."

 - we agree with the reviewer that many RSEs are probably unaware of these code of conducts. However we feel that this is an important aspect for RSEs going forward. They will need at least some basic familiarity with it to support their future work.

 - add sentences to explain why these values are stated explicitly here even though many practioners will learn about these values by following the examples of their peers and mentors.

 - reworded the paragraph on computer-based modeling to improve flow.

 - fixed in markdown version of the paper

 ## Reviewer 1 Feedback 4:

 > can you include a footnote to any digital information on the Paderborn workshop?

 > Section 4.1.2 - there are multiple research software maturity models/frameworks that have been proposed that adapt SWLC. See, for example, M. R. Mundt, W. Burgess and D. M. Vigil, "A Tiered Approach to Scientific Software Quality Practices," in Proceedings of the 2022 Improving Scientific Software Conference (No. NCAR/TN-574+PROC). doi:10.5065/98kd-b491

 > Section 4.1.5 - I recommend adding a note here for "as applicable," e.g., some RSEs at some institutions may not be able to share code publicly because of security or institutional requirements (though they should still use whatever they can for version controlling)

 > Section 4.2.4 - Similar note to 4.1.5; there should be something about "adhering to institutional policies" as well

 > Section 4.4 (Tasks and Responsibilities) - When talking about RSE communities, it might be worth it to reference some of them, e.g., UK-RSE / Soc RSE, deRSE, US-RSE, RSE-AUNZ, RSE Asia, RSSE Africa, etc.

 We thank the reviewer for their feedback! In more detail:

 - We made the original session pads available on zenodo and linked them in the bibliography.

 "Section 4.1.2 - there are multiple research software maturity models/frameworks that have been proposed that adapt SWLC. See, for example, M. R. Mundt, W. Burgess and D. M. Vigil, "A Tiered Approach to Scientific Software Quality Practices," in Proceedings of the 2022 Improving Scientific Software Conference (No. NCAR/TN-574+PROC). doi:10.5065/98kd-b491"

 - We thank the reviewer for this reference and augmented the section on the software life cycle with it.

 "Section 4.1.5 - I recommend adding a note here for "as applicable," e.g., some RSEs at some institutions may not be able to share code publicly because of security or institutional requirements (though they should still use whatever they can for version controlling)

 Section 4.2.4 - Similar note to 4.1.5; there should be something about "adhering to institutional policies" as well"

 - We qualified it in both sections accordingly.

 "Section 4.4 (Tasks and Responsibilities) - When talking about RSE communities, it might be worth it to reference some of them, e.g., UK-RSE / Soc RSE, deRSE, US-RSE, RSE-AUNZ, RSE Asia, RSSE Africa, etc."

 - We thank the referee for this suggestion, and we took then the opportunity to link to the overview page of

 the international RSE council, which lists all national groups, and thereby also will be updated in the future.

 ## Reviewer 1 Feedback: Section 3.1

 >  Section 3.1.1 - this almost might be better if phrased as "Data Security" because that's kind of the crux of the paragraph

 Good point, we have renamed the section.

 >    I notice that "funding methods" is missing from these challenges; there has been a lot of discussion about unreliable funding methods for software and, as a result, RSEs (e.g., https://ieeexplore.ieee.org/stamp/stamp.jsp?arnumber=6886129, https://ieeexplore.ieee.org/stamp/stamp.jsp?arnumber=9470770, https://pure.manchester.ac.uk/ws/portalfiles/portal/54140648/StateOfTheNationReport2017.pdf, https://ieeexplore.ieee.org/stamp/stamp.jsp?arnumber=10071971)

 >    There is also a lack of "RSEs being integrated into existing teams and having to fight that team's culture". Maybe this is too niche to bring up, but it is a challenge that has been spoken about amongst RSE circles (e.g., "How can I get an existing team to change their processes when they are so stuck in their ways?")

 We thank the reviewer for his/her feedback. We now mention the funding problem in the future work section of the paper. We thank the referee for the additional references, Since we don't feel

 that is a challenge that has a stronger influence on the education of an RSE than it would have for any other domain scientist.

 The Integration into teams is now mentioned in the Tasks-and-Responsibilities section.

 ## Reviewer 1 Feedback: Section 5

 > Section 5.2

 >    I disagree with some of the claims here (e.g., license discussion for Bachelor's degree seems like too much). Generally, though, this section is nice. However...

 >    Not all RSEs have PhDs. Most of them do, yes, but many RSEs who do NOT have PhDs are still wildly successful without having gained the proposed skills as PhD students. How do you address that scenario (i.e., where an RSE stops after a Bachelors, Masters, etc.?)

 We agree that a full license discussion would be too much for a Bachelor's degree, we really mean a very basic awareness, e.g. that different licenses exist and may come with obligations like copyleft.

 This would fit in a more general discussion around good scientific practice as a primer to a Bachelor's thesis.

 We expanded on what we mean by awareness to hopefully make this clearer.

 Sections 5.2 is about RSE skills that academic researchers may need without being or ever becoming RSEs,

 so this would not be the place to address this issue. We have expanded the introduction to make this distinction clearer.

 However, we agree that a PhD is not (and should not be) a requirement to work as an RSE and

 as hinted at in the future work section we are working on composing a curriculum for a Master's degree in RSE.

 > Section 5.3 (Project team structures) - there is a gap here: "The single RSE on a team of not-RSEs who does not have a central RSE team connection." These do exist (sadly).

 We have clarified that this type of RSE (alone in a team of non-RSEs) is also meant in the category "Individual RSE (Locally-based)".

 > Section 5.4.1 - formatting weirdness in the Research Skills sub-list (pg 22)

 fixed, thanks for reporting!
